# ChromNet: A Multi‐Task Learning Framework for Cross‐Cell Type Prediction of 3D Chromatin Interactions Using Epigenetic Signals

**DOI:** 10.1002/advs.202508110

**Published:** 2025-10-30

**Authors:** Bin Wang, Shaokai Wang, Liqing Ding, Hongdong Li, Yaohang Li, Jianxin Wang

**Affiliations:** ^1^ Hunan Provincial Key Lab on Bioinformatics School of Computer Science and Engineering Central South University Changsha 410083 China; ^2^ Xiangjiang Laboratory Changsha 410205 China; ^3^ Department of Mathematics Hong Kong University of Science and Technology Clear Water Bay Hong Kong SAR 999077 China; ^4^ Department of Computer Science Old Dominion University Norfolk VA 23529 USA

**Keywords:** cell‐type specificity, chromatin 3D structure, chromatin architecture prediction, epigenetic signals, multi‐task learning

## Abstract

The 3D organization of chromatin plays a fundamental role in gene regulation, cellular function, and disease mechanisms. However, current experimental techniques, such as Hi‐C, remain costly and labor‐intensive, limiting their application in large‐scale and disease‐related studies. To address this challenge, ChromNet is presented, a multi‐task learning framework that integrates epigenetic signals across diverse cell types to enable high‐precision prediction of chromatin architecture. By incorporating noise perturbation and auxiliary classification tasks, ChromNet improves the identification of topologically associating domains (TADs) and cell‐type‐specific chromatin structures, demonstrating superior generalization performance. Notably, ChromNet accurately predicts chromatin interactions in acute myeloid leukemia (AML) samples by leveraging epigenetic signals from both normal and diseased cells, highlighting its potential for studying disease‐associated chromatin remodeling. Across multiple key benchmarks, ChromNet consistently outperforms existing models, providing a robust and cost‐effective solution for large‐scale chromatin conformation studies. This framework enables the exploration of chromatin structural variations across both cell types and disease states, offering new insights into the relationship between 3D genome architecture and gene regulation.

## Graphical abstract

ChromNet enables accurate cross‐cell‐type prediction of chromatin architecture from DNA and epigenetic profiles alone, capturing both conserved and cell‐type‐specific (including disease‐associated) structural features. Its noise‐perturbed multi‐task learning design enhances generalization, enabling precise reconstruction of 3D genome organization even when Hi‐C data for the target cell type are unavailable.

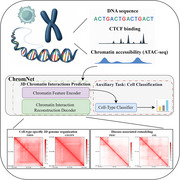

## 1 Introduction

In the nucleus, the genome is intricately folded and organized in a 3D structure, playing a crucial role in gene regulation, cellular function, and disease mechanisms.^[^ ^1^, ^2^ ^]^ This hierarchical organization includes chromatin loops,^[^ ^3^, ^4^ ^]^ topologically associating domains (TADs),^[^ ^5^, ^6^, ^7^ ^]^ and chromatin compartments,^[^ ^8^ ^]^ which facilitate long‐range genomic interactions, thereby influencing gene expression and coordinating gene regulatory networks.^[^ ^9^, ^10^, ^11^ ^]^ Notably, chromatin structure is highly dynamic and undergoes significant remodeling in various diseases, including cancer and developmental disorders.^[^ ^12^, ^13^, ^14^, ^15^, ^16^ ^]^ Understanding these structural changes is essential for elucidating the role of 3D genome organization in disease pathogenesis.

Recent advances in chromatin conformation capture techniques, such as Hi‐C, have greatly enhanced our understanding of 3D chromatin organization. Hi‐C enables genome‐wide mapping of chromatin interactions, uncovering the topological structure of the genome and its role in gene regulatory networks.^[^ ^11^, ^17^ ^]^ Despite its utility, Hi‐C remains constrained by high costs, technical complexity, and extensive experimental requirements, particularly in large‐scale studies involving multiple cell types or disease conditions. The need for large sample sizes and labor‐intensive procedures limits its feasibility for patient‐derived samples and high‐throughput applications.^[^ ^18^, ^19^ ^]^ Moreover, obtaining chromatin interaction data across diverse cell types and disease states solely through experimental approaches remains challenging. These limitations underscore the urgent need for computational models that leverage epigenomic features to accurately predict chromatin architecture, reducing experimental dependency while expanding the scope of genomic research.^[^ ^20^ ^]^

In this context, deep learning has emerged as a powerful tool for modeling complex biological systems, significantly reducing reliance on experimental chromatin conformation data.^[^ ^20^, ^21^, ^22^, ^23^, ^24^ ^]^ Several deep learning methods have been applied to predict chromatin structure, including Akita,^[^ ^25^ ^]^ DeepC,^[^ ^26^ ^]^ Orca,^[^ ^27^ ^]^ Epiphany,^[^ ^28^ ^]^ and C.Origami.^[^ ^29^ ^]^ These methods leverage DNA sequences and epigenetic data to predict chromatin interactions, advancing the field of 3D genome modeling.

Akita emphasizes the role of CTCF binding sites in chromatin architecture,^[^ ^25^ ^]^ but it does not fully account for cell‐type‐specific differences, limiting its generalization across multiple cell types. DeepC employs transfer learning to predict chromatin folding;^[^ ^26^ ^]^ however, its performance on novel cell types remains suboptimal. Orca explores multiscale chromatin structure based on genomic sequence,^[^ ^27^ ^]^ yet its complexity restricts its application in large‐scale studies. Epiphany predicts Hi‐C contact maps using 1D epigenomic signals and achieves strong performance on specific datasets,^[^ ^28^ ^]^ but struggles with cross‐cell‐type generalization. The most advanced model to date, C.Origami, combines DNA sequences and epigenetic features for chromatin structure prediction.^[^ ^29^ ^]^ It outperforms earlier models like Akita, DeepC, and Orca in terms of prediction accuracy on specific cell types. However, C.Origami also faces limitations when generalizing across different cell types, as it falls short in capturing chromatin structural variations between cell types. In summary, these methods primarily rely on features from individual cell types, restricting their generalization across diverse cellular contexts. Given that chromatin architecture varies across cell types and is dynamically remodeled in diseases like cancer, there is a critical need for an improved framework that integrates multi‐cell‐type epigenomic signals to enhance predictive accuracy. Such a model would not only broaden the utility of deep learning for 3D genome modeling but also offer novel insights into the role of chromatin remodeling in disease mechanisms.

To overcome these limitations, we propose ChromNet, a multi‐task learning framework that leverages both conserved and cell‐type‐specific chromatin structures to enhance model generalization. While existing models struggle with cross‐cell‐type predictions, ChromNet introduces an innovative strategy by perturbing the epigenetic signals of the target cell type with Gaussian noise. This approach enhances the model's robustness to variations in epigenetic signals, where differences in CTCF and ATAC‐seq signals, despite sharing the same DNA sequence, influence chromatin interactions. By integrating noise‐perturbed epigenetic signals from the target cell type with epigenetic features (CTCF and ATAC‐seq) from other cell types, the model effectively captures both conserved and cell‐type‐specific chromatin characteristics. This enables ChromNet to learn the relationship between epigenetic signals and chromatin structure, thereby improving its cross‐cell‐type prediction accuracy. The inclusion of a classification task further enhances its ability to distinguish cell‐type‐specific patterns. Notably, in acute myeloid leukemia (AML) samples, ChromNet reconstructs chromatin conformations with high fidelity by integrating epigenetic signals from both normal and malignant cells, demonstrating its potential in studying disease‐associated chromatin remodeling. ChromNet achieves superior performance across multiple key metrics, exhibiting stronger correlations with experimental Hi‐C data and effectively capturing chromatin interactions in both physiological and pathological contexts. ChromNet provides a robust and cost‐effective framework for large‐scale chromatin structure prediction, offering new avenues to explore chromatin structural variations across different cell types and disease states. This approach lays a solid foundation for deciphering the interplay between 3D genome organization and gene regulation, particularly in disease progression and targeted therapy research.

## 2 Results

### 2.1 Overview of ChromNet

**Figure 1:**
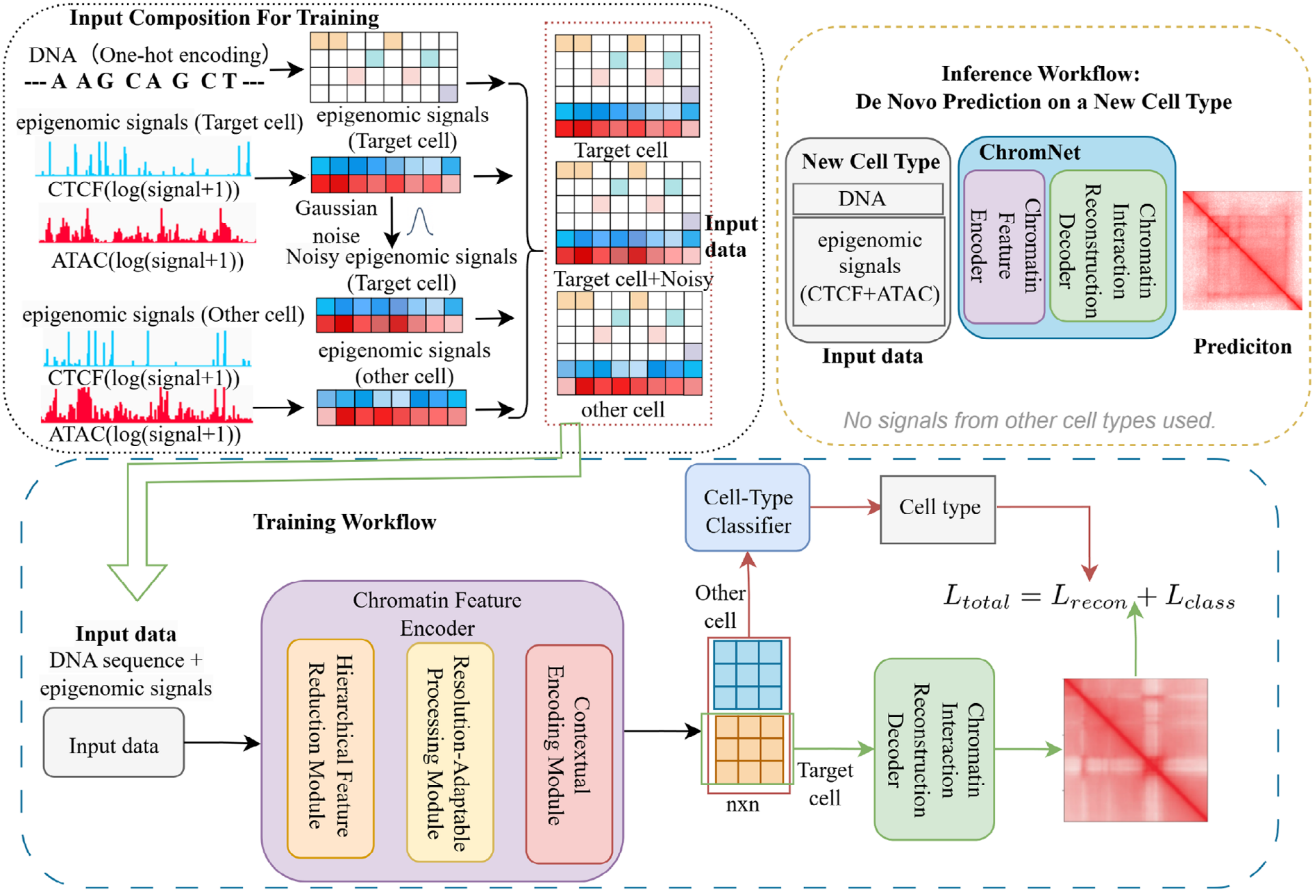
The architecture of ChromNet. ChromNet is a multi‐task learning framework for predicting 3D chromatin interactions across cell types. During training, the model receives the DNA sequence and epigenetic signals (CTCF and ATAC‐seq) from a target cell, including both original and Gaussian noise‐perturbed signals, each as separate input samples. Epigenetic signals from another cell type are also included to enhance generalization. The encoder output is fed into two branches: a chromatin interaction reconstructor supervised by Hi‐C, and a cell‐type classifier. During inference, only the DNA and epigenetic signals from a new cell type are used. No noisy signals or signals from other cell types are included.

ChromNet is a multi‐task learning framework designed to enhance chromatin interaction prediction across different cell types. The model integrates nucleotide‐resolution DNA sequence features with cell‐type‐specific epigenetic signals (CTCF and ATAC‐seq) as inputs to predict Hi‐C interaction matrices. To improve generalization, ChromNet applies Gaussian noise perturbation to the epigenetic signals of the target cell type, enhancing the model's robustness to epigenetic variations and improving cross‐cell‐type adaptability. Additionally, the model incorporates epigenetic signals from other cell types during feature encoding, enabling the capture of both conserved and cell‐type‐specific chromatin structures. The architecture of ChromNet consists of three key components. First, the Chromatin Feature Encoding Unit extracts multi‐scale features from input signals and dynamically adjusts feature resolution to accommodate chromatin interactions at different scales. These features are then passed to the Chromatin Interaction Reconstruction Unit, which predicts the 3D chromatin structure. Furthermore, a Cell‐Type Classification Unit learns epigenetic differences across cell types to optimize feature representation. ChromNet's overall architecture is illustrated in **Figure** **1**. A detailed description of the model structure and parameters is provided in Figure S1 (Supporting Information). The primary task of ChromNet is chromatin interaction prediction, while epigenetic perturbation and multi‐cell‐type feature integration mechanisms contribute to its adaptability and generalization across different cell types.

### 2.2 Performance Evaluation of ChromNet on Training Cell Types

We presented a detailed comparison of ChromNet's performance against C.Origami and Epiphany in predicting Hi‐C interaction matrices on known cell types. Given that existing studies have shown C.Origami significantly outperforms other models like Atika, DeepC, and Orca, our focus here is on comparing ChromNet with C.Origami and Epiphany. Since each method employs different ground truth data, transformations, and resolutions, direct comparisons among all three are not feasible. For C.Origami, we retrained the method using the same data split and resolution (8,192 bp) as ChromNet, as both methods share consistent ground truth data. This ensures a fair evaluation of the two methods. For Epiphany, ChromNet was trained using the Resolution‐Adaptable Processing Module to align with Epiphany's pre‐processed Hi‐C interaction matrix data (Obs/Exp) and original data split, at a resolution of 10 kb. Despite these variations in ground truth and data preprocessing, we independently assess the performance of each method to highlight ChromNet's ability to handle diverse input formats and resolutions, further demonstrating its advantages in predicting 3D chromatin interactions from DNA sequences and epigenetic signals.

**Figure 2:**
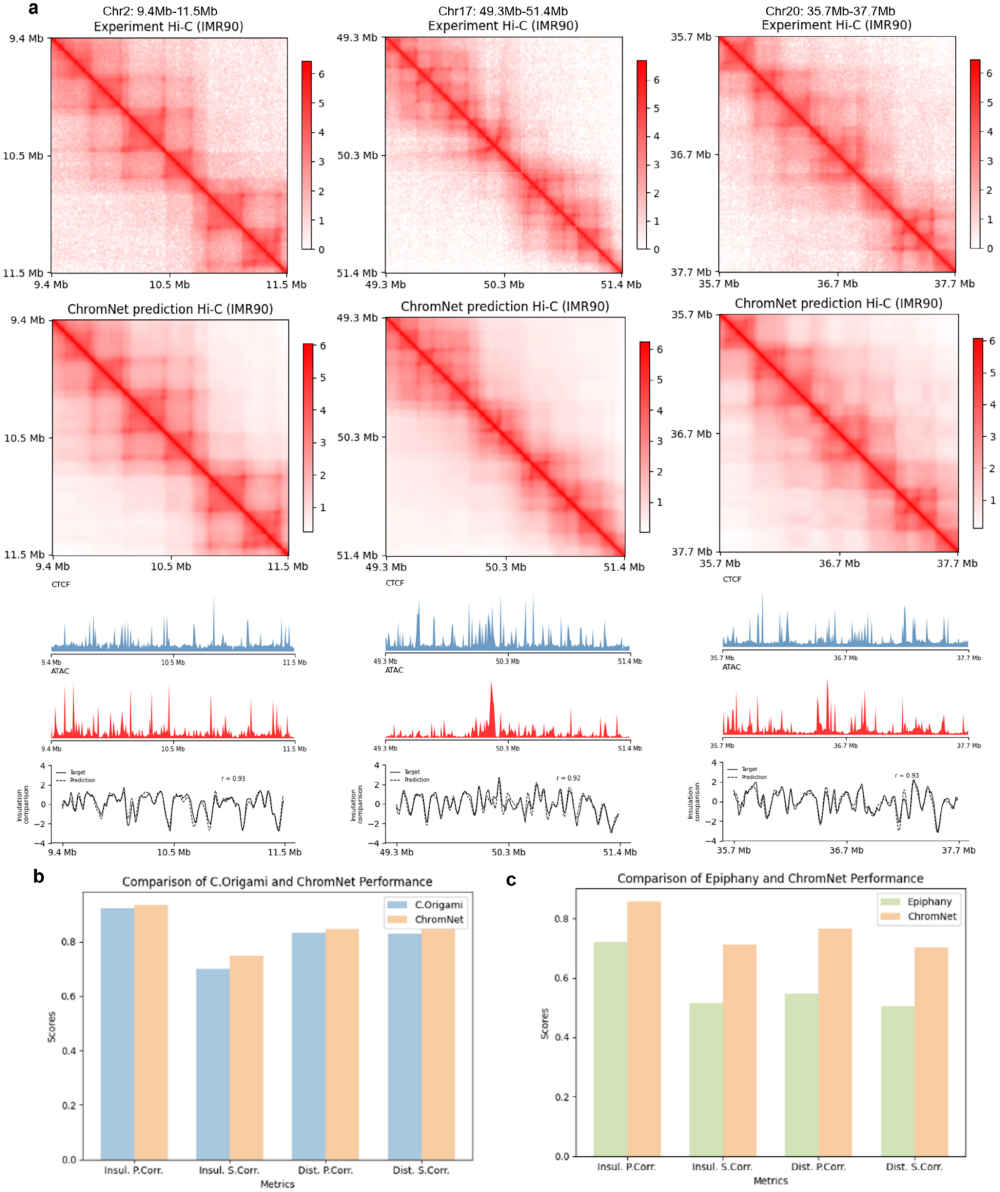
ChromNet predictions across data partitions and comparative evaluation with baseline models. a) ChromNet predictions on representative regions from training, validation, and test sets. Each region shows, from top to bottom: experimental Hi‐C, predicted Hi‐C, CTCF signal, ATAC‐seq signal, and insulation score curves (z‐score normalized), with Pearson correlation (r) labeled. b) Comparison with C.Origami using shared ground truth and 8,192 bp resolution, evaluated by insulation and distance‐based Pearson and Spearman correlations. c) Comparison with Epiphany based on its original data split and 10 kb resolution. ChromNet predictions are adjusted using the Resolution‐Adaptable Processing Module.

To comprehensively assess the generalizability and robustness of ChromNet across different genomic regions, we first examined its predictive performance on the training, validation, and test partitions (**Figure** **2**a). The results demonstrate that ChromNet faithfully reconstructs key structural features observed in experimental Hi‐C contact maps across all partitions, including well‐defined domain boundaries and enriched interaction regions. On the test set, in particular, the predicted insulation scores show strong agreement with the ground truth, achieving Pearson correlation coefficients as high as 0.96, indicating exceptional boundary‐detection performance.

We further benchmarked ChromNet against two state‐of‐the‐art models, C.Origami and Epiphany. As shown in Figure 2b, ChromNet consistently outperforms C.Origami across all evaluation metrics, with especially notable improvements in the Spearman correlation of the insulation score, reflecting its enhanced sensitivity to relative boundary strength. In Figure 2c, we compared ChromNet with Epiphany using its original data split and pre‐processed Obs/Exp Hi‐C matrices. ChromNet again demonstrated substantial superiority in both insulation score and distance‐stratified correlations (Pearson and Spearman), highlighting the effectiveness of its resolution‐adaptive module in handling interaction signals at multiple genomic scales and further boosting overall prediction performance.

### 2.3 ChromNet‐Based De Novo Prediction of Chromatin Organization Across Cell Types

We compared the performance of ChromNet and C.Origami in the cross‐cell‐type de novo prediction task. The models were first trained on the IMR‐90 cell line and subsequently evaluated on unseen target cell types, including K562, GM12878, and HCT116. Unlike predictions for known cell types, cross‐cell‐type predictions are more challenging, as the methods must infer the 3D chromatin structure based on DNA sequences and epigenetic signals (such as CTCF and ATAC‐seq) without using Hi‐C interaction matrices as supervision. Considering the limited performance of Epiphany on the trained cell types, we did not choose to perform a comprehensive comparison with ChromNet in this experiment, focusing instead on comparisons with C.Origami. This choice allows us to better evaluate the generalization capability of ChromNet across different cell types and understand its advantages in cross‐cell‐type predictions more effectively.

**Figure 3:**
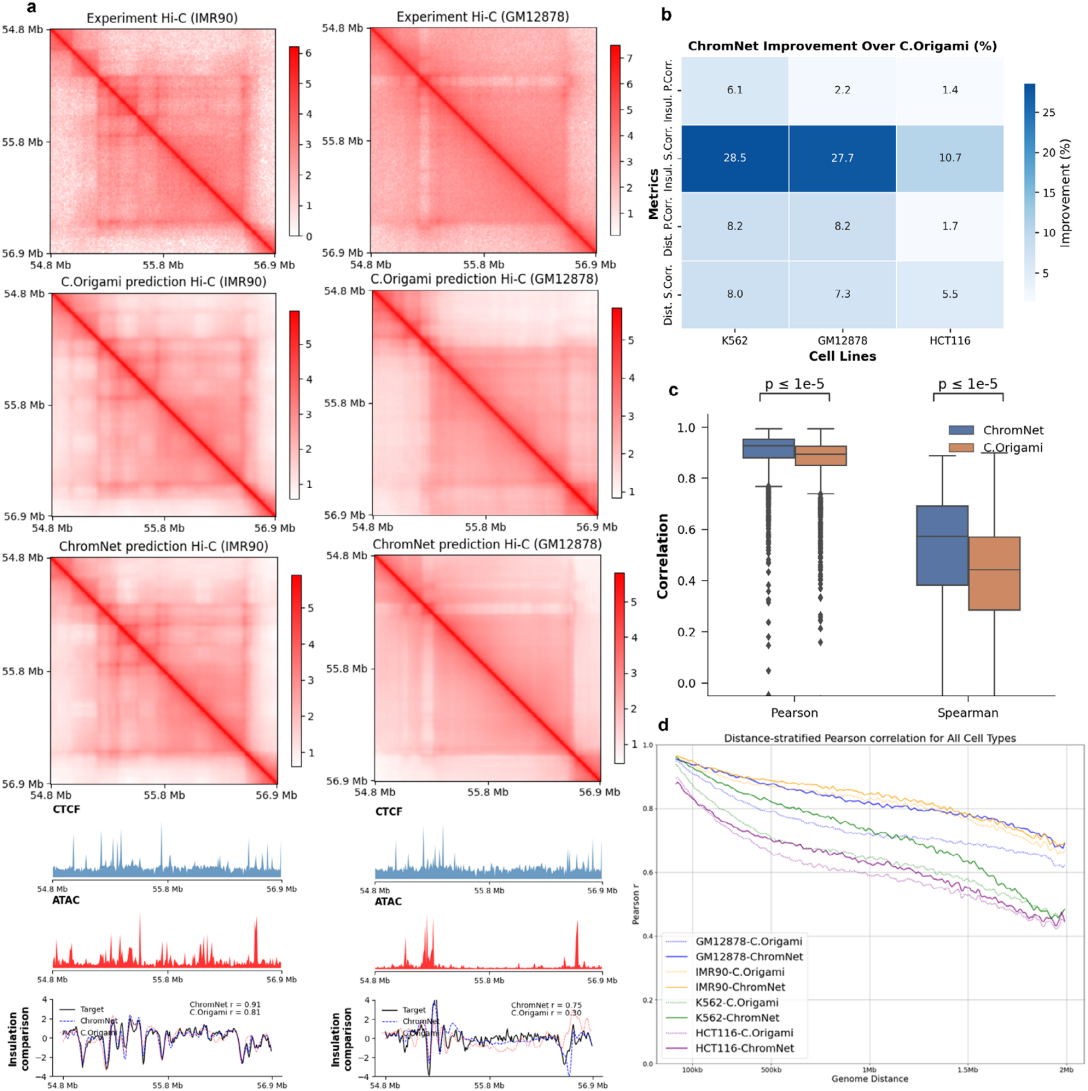
Performance of ChromNet in cross‐cell‐type prediction. a) Predictions on a representative genomic region from IMR‐90 and GM12878. For each cell type, shown from top to bottom are: experimental Hi‐C, C.Origami prediction, ChromNet prediction (all at 8,192 bp), followed by CTCF, ATAC‐seq signals, and z‐score normalized insulation scores. Pearson correlation between predicted and experimental insulation scores is reported. b) Heatmap showing ChromNet's percentage improvement over C.Origami on held‐out cell types (K562, GM12878, HCT116), evaluated using four metrics: Insul. P.Corr., Insul. S.Corr., Dist. P.Corr., and Dist. S.Corr. Improvement is computed as (ChromNet ‐ C.Origami) / C.Origami × 100%, with values annotated. c) Boxplots comparing insulation score correlations across the same cell types. ChromNet consistently outperforms C.Origami in both Pearson and Spearman correlations (paired t‐test). Error bars indicate interquartile ranges. d) Pearson correlation between predicted and experimental Hi‐C maps stratified by genomic distance within 2 Mb. Each curve represents a model–cell type pair.

We evaluated the performance of ChromNet and C.Origami in Hi‐C prediction tasks across different cell types. **Figure** **3**a presents the experimental Hi‐C and its predicted results, demonstrating that ChromNet reconstructs local and mid‐range chromatin interactions more accurately while effectively reducing artifacts, leading to greater overall consistency with experimental data. Additional visualizations of the prediction results are provided in Figure2 and S3 (Supporting Information), further highlighting the performance differences between the two models. These results underscore ChromNet's advantages in cross‐cell‐type prediction tasks.

To further quantify the performance differences between ChromNet and C.Origami, we evaluated both models using multiple correlation‐based metrics, including Pearson and Spearman correlations of insulation scores, as well as distance‐stratified Pearson and Spearman correlations (Figure 3b; Table S4, Supporting Information). Across all tested cell types (K562, GM12878, and HCT116), ChromNet consistently outperformed C.Origami. The improvements were particularly notable for Spearman correlation of insulation scores, with increases of 28.5% in K562 and 27.7% in GM12878. Boxplots in Figure 3c further demonstrate that, across all cross‐cell‐type predictions, ChromNet achieves statistically significant improvements in the Pearson and Spearman correlations of insulation scores (paired t‐test, p < 1e–5). Violin plots for insulation score correlations across individual cell types are shown in Figure S4c,d.

We further examined distance‐stratified prediction accuracy (Figure 3d and Figure S4b, Supporting Information). ChromNet maintained superior performance across all tested cell types, with particularly strong improvements in short‐ and medium‐range interaction regions (<1.5 Mb). This observation is consistent with fundamental principles of chromatin folding, where local interactions play a key role in gene regulation, further supporting the accuracy and generalizability of ChromNet for cross‐cell‐type 3D chromatin structure prediction.

To investigate the impact of different training strategies on ChromNet's predictive performance, we conducted a comprehensive ablation study. The study evaluated both intra‐cell type performance (IMR‐90) and cross‐cell type predictions (GM12878, K562, HCT116), comparing various model variants. ChromNet_base served as the baseline, trained solely on IMR‐90 epigenetic signals without incorporating additional cell‐type data or noise perturbation. ChromNet_noise introduced Gaussian noise to IMR‐90's CTCF and ATAC‐seq signals during training to simulate variability and improve the model's generalization across cell types. The performance of each model was systematically evaluated across the test cell types, focusing on Pearson and Spearman correlations for both insulation score and distance‐stratified metrics.

Introducing noise perturbation markedly improved model robustness. Compared to ChromNet_base, ChromNet_noise achieved superior performance across most cell types, indicating that mild noise augmentation facilitates more stable learning of key chromatin features (Table S5, Supporting Information). Although it demonstrates stronger structural awareness, its generalization across cell types remains limited. Building upon this, the integration of epigenomic signals from multiple cell types led to substantial improvements across all evaluation metrics, with particularly pronounced gains in insulation score correlations. This highlights ChromNet's enhanced adaptability to diverse chromatin landscapes and its robust cross‐cell‐type generalization. In contrast, ChromNet_base consistently underperformed across all cell types, underscoring the limitations of models trained on a single cell‐type background.

Beyond training strategy analysis, we conducted a structural ablation study to examine the importance of key architectural components in ChromNet. To this end, we replaced ChromNet's encoder, decoder, and sequence modeling modules with their counterparts from C.Origami, generating three simplified variants (ChromNet_Enc, ChromNet_Dec, ChromNet_Tran). As shown in Table S6 (Supporting Information), all components contributed substantially to model performance, underscoring the importance of tailored architectural designs for accurate and generalizable chromatin structure prediction. In particular, the performance drop observed in ChromNet_Tran highlights that LSTM is better suited for capturing the directional and sparse nature of genomic epigenetic signals. Unlike Transformer's attention mechanism, which may be sensitive to sparse or noisy input such as low‐frequency CTCF peaks, LSTM enables more stable integration of localized signal patterns across genomic regions.

To further investigate cross‐cell‐type prediction effectiveness, we compared the performance of ChromNet and ChromNet (GM12878) in detail. While both models were trained under the multi‐task learning framework, ChromNet (GM12878) used GM12878 epigenetic signals in place of K562 to examine the impact of varying cell‐type signals on model generalization ability, as shown in Table S7 (Supporting Information). Results indicate that incorporating epigenetic signals from the specific cell type intended for de novo prediction significantly enhances chromatin structure prediction accuracy, underscoring the critical role of leveraging biologically similar cell‐type signals to improve cross‐cell‐type predictions. Notably, this improvement is achieved even without using Hi‐C labels from the new cell type during training, indicating that epigenetic inputs alone can provide sufficient context for effective generalization.

We systematically explored the impact of Gaussian noise injection during training to enhance model robustness and cross‐cell‐type generalization. Initial experiments identified a fixed standard deviation of 0.1 applied uniformly to both CTCF and ATAC‐seq signals (after log transformation) as optimal, as higher noise levels degraded performance by obscuring critical cell‐type‐specific signals (Table S8, Supporting Information).

To further evaluate noise injection strategies, we compared ChromNet with fixed SD = 0.1 against three dynamically scaled noise schemes based on the genome‐wide means of CTCF and ATAC signals in IMR‐90. As summarized in Supplementary Table S9, dynamically scaled noise slightly reduced insulation score correlations while sometimes improving distance‐stratified correlations, indicating distinct trade‐offs. These results suggest that both fixed and dynamic noise strategies can be effective depending on whether precise TAD boundary prediction or broader chromatin architecture modeling is prioritized.

These findings demonstrate that introducing noise perturbation simulates epigenetic variability, helping the model better capture both conserved and cell‐type‐specific features of chromatin architecture. By incorporating epigenetic signals from multiple cell types, ChromNet outperforms existing models on various metrics, further validating the necessity of multi‐cell‐type epigenetic integration for robust cross‐cell type chromatin structure prediction.

To further interpret how the auxiliary classifier facilitates cross‐cell‐type prediction, we analyzed its internal feature representations on the test set and visualized them using t‐SNE, which revealed distinct clusters corresponding to IMR‐90 and K562 samples (Figure S5a, Supporting Information). However, a subset of samples remained intermingled in the t‐SNE space; these corresponded to genomic regions with low Hi‐C contact frequencies and near‐zero CTCF and ATAC signals, likely lacking active regulatory information and potentially explaining the classifier's reduced ability to confidently distinguish them. Saliency scores computed with Integrated Gradients (IG)^[^ ^30^ ^]^ revealed prominent peaks overlapping several K562‐upregulated genes associated with leukemia biology (Figure S5b, Supporting Information).

### 2.4 The Role of CTCF and ATAC in Hi‐C Interaction Prediction

To investigate the contribution of different epigenetic features, we trained two models: one using only CTCF binding data (ChromNet_onlyCTCF) and another using only ATAC‐seq chromatin accessibility data (ChromNet_onlyATAC). Model performance was evaluated across multiple cell types using insulation score Pearson/Spearman correlations and distance‐stratified Pearson/Spearman correlations. More detailed results are provided in Table S10 (Supporting Information).

**Figure 4:**
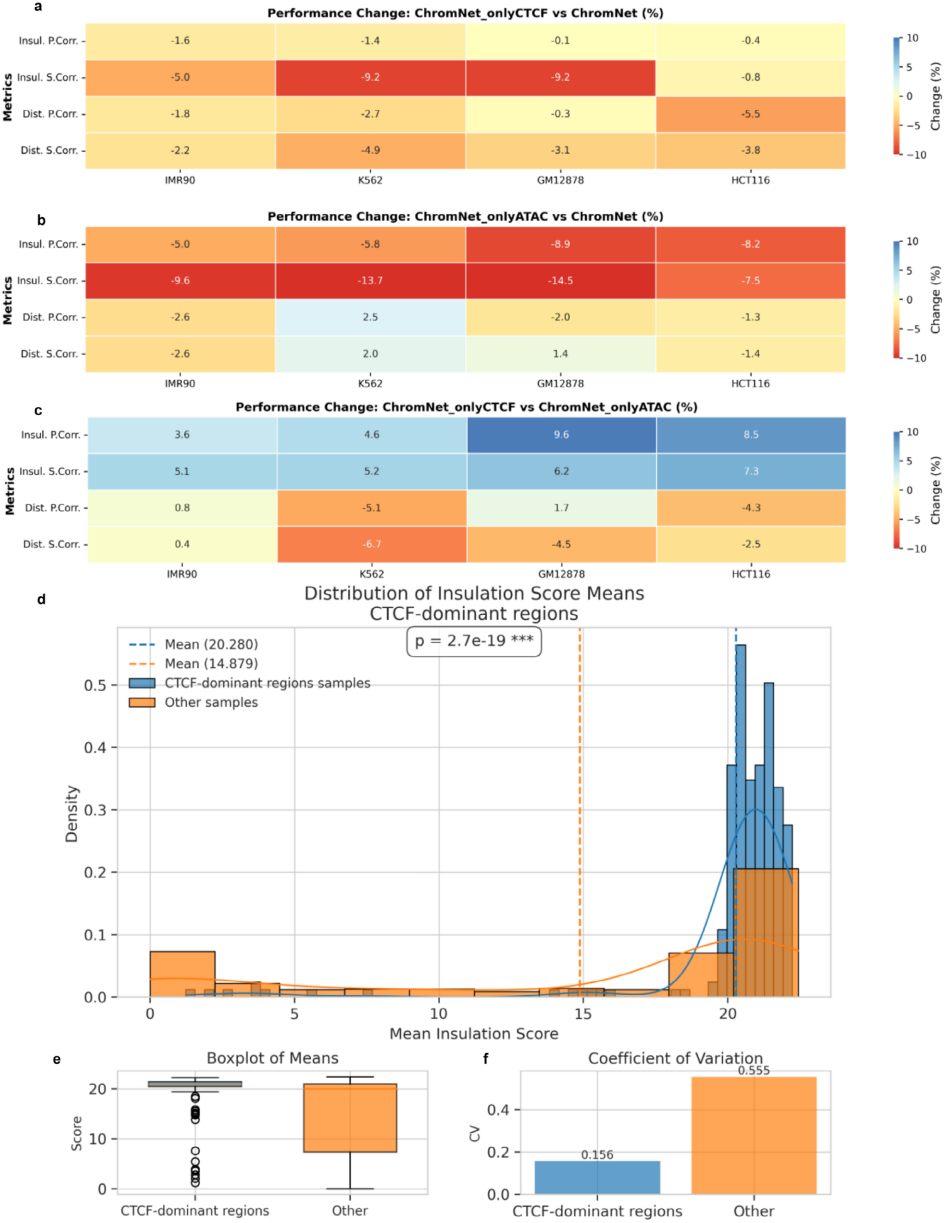
Dissecting the role of CTCF and ATAC signals in Hi‐C interaction prediction. a) Performance of ChromNet_onlyCTCF relative to ChromNet, computed as (ChromNet_onlyCTCF ‐ ChromNet) / ChromNet x 100%. b) Performance of ChromNet_onlyATAC relative to ChromNet, computed as (ChromNet_onlyATAC ‐ ChromNet) / ChromNet x 100%. c) Performance difference between ChromNet_onlyCTCF and ChromNet_onlyATAC, computed as (ChromNet_onlyCTCF ‐ ChromNet_onlyATAC) / ChromNet_onlyATAC x 100%. Positive (blue) and negative (red) values indicate relative gains or losses. d) Density plot of mean insulation scores in CTCF‐dominant (blue) versus. other regions (orange), with vertical dashed lines marking group means (p = 2.7e‐19). e) Boxplot comparing insulation score distributions; CTCF‐dominant regions show more concentrated scores. f Coefficient of variation (CV) comparison: 0.156 for CTCF‐dominant vs. 0.555 for other regions, indicating higher variability in the latter.

Removing ATAC‐seq signals (**Figure** **4**a) led to a moderate performance decline across most metrics and cell types. In IMR‐90, insulation score Pearson and Spearman correlations decreased by 1.6% and 5.0%, respectively, while distance‐stratified Pearson and Spearman correlations declined by 1.8% and 2.2%. Similar trends were observed in K562 and GM12878, with the largest drop seen in the insulation score Spearman correlation (–9.2%). These results suggest that although CTCF captures major structural features, ATAC‐seq contributes additional cell‐type–specific information, particularly in capturing local insulation strength.

In contrast, removing CTCF binding signals (Figure 4b) caused a more substantial performance degradation. For instance, in IMR‐90, insulation score Pearson and Spearman correlations dropped by 5.0% and 9.6%, respectively; in GM12878, Spearman correlation declined by 14.5%. Across all four cell types, the performance loss was consistently larger than that caused by removing ATAC‐seq, underscoring CTCF's critical role in defining chromatin boundaries and maintaining structural insulation.

A direct comparison between ChromNet_onlyCTCF and ChromNet_onlyATAC (Figure 4c) revealed differing trends across correlation metrics. ChromNet_onlyCTCF outperformed ChromNet_onlyATAC in insulation score correlations across most cell types. However, for distance‐stratified correlations, ChromNet_onlyATAC exhibited superior performance in some cases.

To systematically analyze such cases, CTCF‐dominant regions were defined, where ChromNet_onlyCTCF exhibited higher insulation score correlations but lower distance‐stratified correlations compared to ChromNet_onlyATAC. The distribution of mean insulation scores within CTCF‐dominant regions is shown in Figure 4d. A significant difference was observed between two groups (p = 2.7e‐19), with CTCF‐dominant regions displaying a higher mean insulation score (≈20.28) compared to other genomic regions (≈14.88).

Further statistical analysis indicates that CTCF‐dominant regions exhibit lower variability in insulation scores, with a coefficient of variation (CV) of only 0.156, compared to 0.555 for other samples (Figure 4e). Additionally, the boxplot (Figure 4f) shows that CTCF‐dominant regions have a higher mean insulation score with a more concentrated distribution, suggesting that these regions have more stable insulation properties. This observation is consistent with the density plot above, where CTCF‐dominant regions exhibit higher insulation scores with less fluctuation.

These results indicate that ChromNet_onlyCTCF performs better in predicting chromatin topological boundaries, while ChromNet_onlyATAC provides advantages in capturing global chromatin topology changes. Specifically, CTCF‐dominant regions tend to exhibit higher insulation scores with lower variability, suggesting that CTCF binding signals may play a primary role in establishing chromatin insulation structures in these regions. In contrast, in more dynamically structured chromatin regions, chromatin accessibility signals from ATAC‐seq may be more informative, leading to better performance in distance‐stratified correlation analyses.

Overall, different correlation metrics reveal distinct aspects of chromatin topology prediction, and CTCF binding signals and ATAC‐seq accessibility provide complementary information. This suggests that integrating both epigenetic features enables a more comprehensive understanding of chromatin structural changes across different cell types, ultimately enhancing the predictive accuracy and generalizability of ChromNet.

### 2.5 ChromNet for Disease‐Associated Chromatin Architecture Analysis

This study further evaluates ChromNet's ability to predict chromatin structures under pathological conditions, focusing on acute myeloid leukemia (AML) datasets.^[^ ^15^ ^]^ Unlike previous cross‐cell‐type analyses, which primarily assessed the model's generalization across different common cell lines (e.g., IMR‐90, K562, and GM12878), this study presents a fundamentally different challenge. AML is characterized by widespread chromatin remodeling, which disrupts the gene regulatory network. Predicting chromatin structures in AML cells using a method trained on healthy peripheral blood mononuclear cells (PBMCs) poses a significant challenge. By integrating epigenetic signals from both normal and malignant cells, ChromNet effectively captures disease‐associated chromatin structural changes, making it a valuable tool for studying 3D genome organization in leukemia.

**Figure 5:**
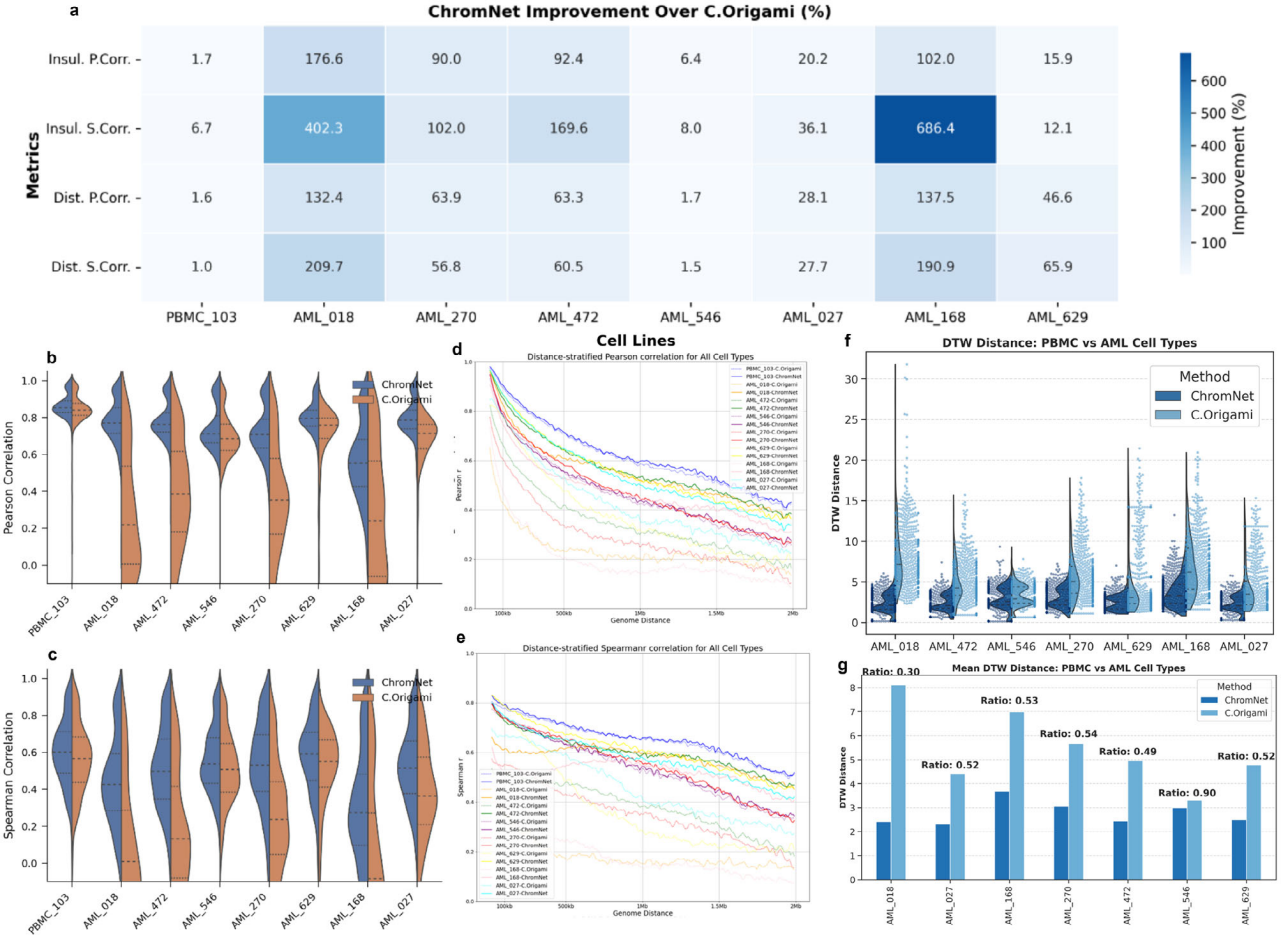
Performance comparison of ChromNet for AMLs. a) Quantitative improvements of ChromNet over C.Origami across PBMC_103 and AML samples. Heatmap showing the percentage improvement of ChromNet relative to C.Origami in PBMC_103 and AML samples. Percent improvement is computed as (ChromNet ‐ C.Origami) / C.Origami × 100%, with values annotated in each cell. b, c) Violin plots showing the distribution of insulation score correlations (Pearson and Spearman, respectively) for ChromNet (blue) and C.Origami (orange) across PBMC_103 and AML samples. d, e) Distance‐stratified Pearson and Spearman correlation curves for PBMC_103 and AML samples, illustrating the predictive performance of ChromNet and C.Origami at varying genomic distances. f) The violin plot illustrates the distribution of DTW distances between PBMC and different AML samples, comparing the performance of ChromNet and C.Origami. Overall, ChromNet exhibits lower DTW distances than C.Origami, suggesting that it captures the variation in insulation scores between PBMC and AML cell types more effectively. g) The bar plot presents the mean DTW distances between PBMC and each AML samples, along with the ChromNet‐to‐C.Origami distance ratio (Ratio = ChromNet / C.Origami).

ChromNet consistently outperforms C.Origami across all evaluation metrics in both PBMC and AML samples (**Figure** **5**a; Table S11, Supporting Information). Although the improvement in PBMC_103 is relatively modest—for instance, the Pearson and Spearman correlations of the insulation score increase by only 1.7% and 6.7%, respectively—the performance gains in AML samples are substantial. For example, in AML_018, insulation score Spearman correlation improves by 402.3%, and distance‐stratified Spearman correlation by 209.7%. Similarly, in AML_168, the improvements reach 686.4% and 190.9%, respectively. This consistent trend across multiple AML samples highlights ChromNet's superior ability to capture leukemia‐specific chromatin architectural features. These results suggest that training with auxiliary cell types sharing epigenomic or lineage similarity with the prediction targets enhances generalization to unseen disease samples, underscoring the importance of informed auxiliary cell‐type selection in cross‐cell modeling.

To further illustrate the predictive performance of ChromNet, we visualized the Hi‐C prediction results for PBMC_103 and two AML samples with significant performance differences (as shown in Figure S6, Supporting Information). Among them, AML_018 exhibited a large performance gap between ChromNet and C.Origami, whereas AML_546 showed only a minor difference. These visualizations intuitively demonstrate ChromNet's capability to reconstruct chromatin interactions under both normal and pathological conditions.

As illustrated in Figure 5b,c, the violin plots provide an intuitive visualization of the distribution of insulation score correlations across PBMC and AML cells. In PBMC_103 cells, both methods exhibit relatively high correlation distributions, but ChromNet has a higher median and a more concentrated distribution, suggesting more stable predictions. However, in AML cells, the advantage of ChromNet becomes more evident, characterized by higher correlation distributions and lower variance, whereas C.Origami shows lower correlations with greater performance variability across different cells. By computing the Cohen's d effect size for the Pearson and Spearman correlation coefficients of the insulation score, as shown in Figure S7 (Supporting Information), we systematically evaluated the performance differences between ChromNet and C.Origami across various cell types. The results indicate that in normal cells (e.g., PBMC_103), the performance difference between the two models is minimal (Cohen's d < 0.2), suggesting comparable predictive capabilities. However, in acute myeloid leukemia (AML) samples, ChromNet demonstrates a significant advantage, with Cohen's d values generally exceeding 0.8. Notably, in samples such as AML_018 and AML_472, Cohen's d surpasses 1.5, indicating that ChromNet more accurately captures disease‐associated chromatin structural changes. Furthermore, the distance‐stratified correlation curves in Figure 5d,e reveal predictive performance across different genomic distances. Overall, as genomic distance increases, Pearson and Spearman correlations decline across all cell types. However, ChromNet maintains consistently higher correlations, particularly for long‐range genomic interactions (>1Mb), where C.Origami's performance degrades more substantially. These results suggest that ChromNet is better at capturing long‐range chromatin interactions, leading to more stable insulation score predictions across different cell types. This capability is particularly crucial in AML cells, where leukemia‐associated chromatin remodeling may hinder the generalization of C.Origami, while ChromNet remains robust to such structural variations.

To comprehensively evaluate the ability of ChromNet and C.Origami in predicting chromatin topology changes during PBMC‐to‐AML cell type transition, we focused on the dynamic changes of insulation score. The insulation score reflects the strength of topologically associating domains (TADs) boundaries, and its dynamic pattern can reveal chromatin remodeling during PBMC‐to‐AML transformation. To quantify the agreement between model predictions and experimental data, we performed Dynamic Time Warping (DTW) distance analysis.^[^ ^31^ ^]^ DTW measures the similarity between the experimentally observed PBMC‐AML insulation score dynamics and those predicted by ChromNet and C.Origami. A lower DTW distance indicates a higher degree of concordance between the predicted and experimental trajectories, reflecting a superior ability to model chromatin topology dynamics.

As illustrated in Figure 5f,g, ChromNet significantly outperforms C.Origami in predicting insulation score dynamics between PBMC and AML cell types. ChromNet consistently achieves lower DTW distances, with reduced variance and fewer extreme outliers, highlighting its superior ability to model chromatin structural changes in disease contexts. In contrast, C.Origami exhibits higher and more dispersed DTW distances, suggesting weaker generalization across AML samples. These findings underscore ChromNet's potential as a robust computational framework for predicting chromatin topology alterations in disease states.

These findings suggest that ChromNet exhibits a stronger modeling capability for chromatin structure changes in disease states such as leukemia, while its advantage is less pronounced in normal cell types. This further supports the potential application of ChromNet in studying disease‐associated chromatin remodeling, offering new insights into the epigenetic mechanisms of cancer and other diseases.

### 2.6 ChromNet‐Based Analysis of 3D Chromatin Perturbations

In this experiment, we aimed to predict disturbances in 3D chromatin architecture caused by epigenomic alterations, specifically focusing on structural changes induced by the deletion of CTCF binding sites. We evaluated the capability of ChromNet to predict these structural changes after perturbing the CTCF input tracks, where the perturbation involved the removal of CTCF peaks.

**Figure 6:**
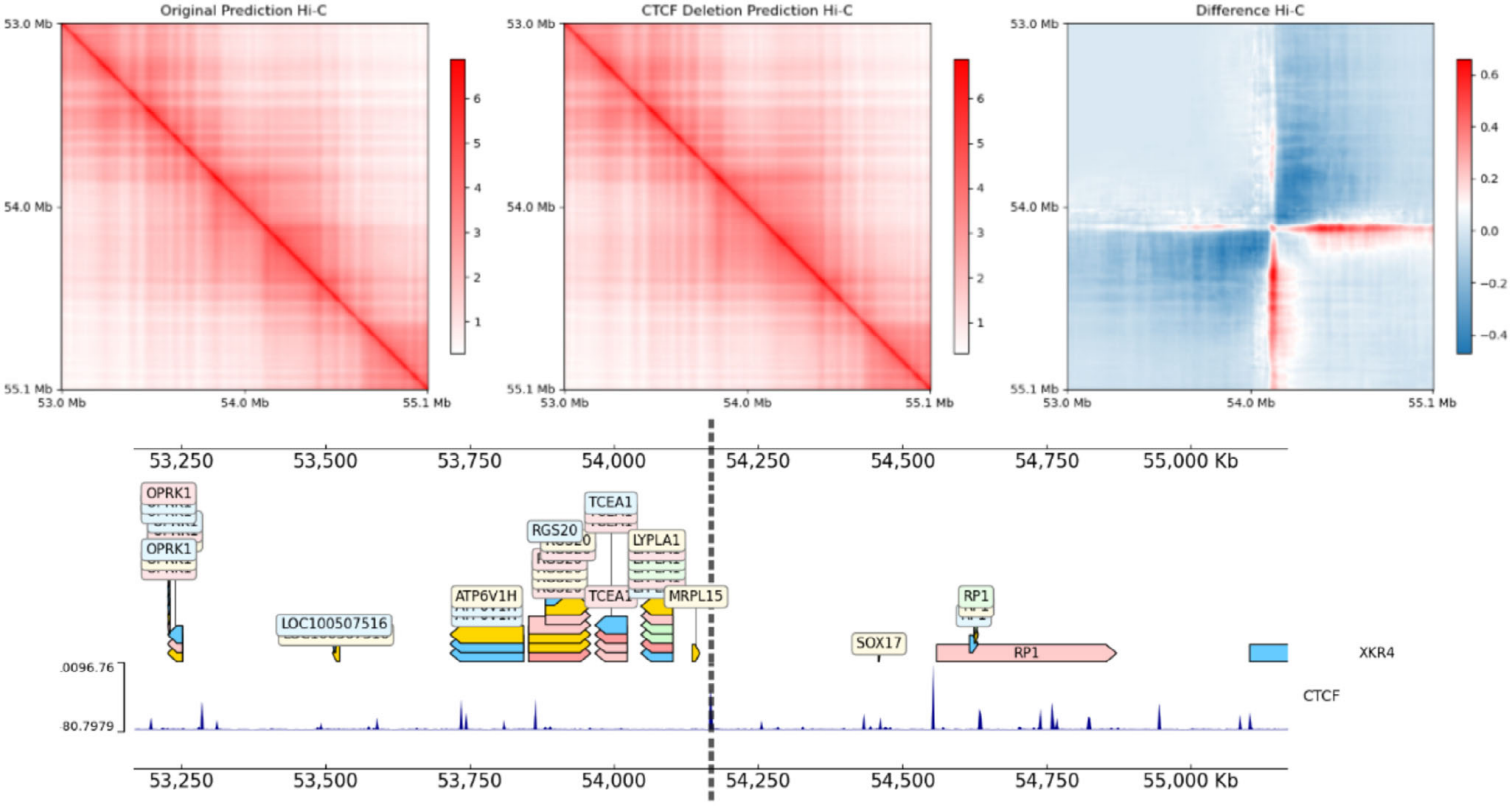
Visualization of 3D Chromatin Structure Perturbations Following CTCF Binding Site Deletion at the SOX17 Locus. The top row presents Hi‐C contact maps: the left panel shows the original predicted Hi‐C interaction matrix, the middle panel depicts the Hi‐C prediction after CTCF binding site deletion, and the right panel displays the difference matrix, calculated as the original Hi‐C prediction minus the CTCF‐deletion Hi‐C prediction. This highlights chromatin interaction changes due to CTCF deletion (red for decreased interactions and blue for increased interactions). The bottom panel provides genomic context, including gene annotations, CTCF binding sites, and ChIP‐seq signal tracks at the corresponding genomic region.

The experiment was conducted using the H1‐hESC cell line, where a CTCF binding site was deleted near the SOX17 locus at hg38 (chr8:54,165,000–54,170,000), with results illustrated in **Figure** **6**. Previous studies^[^ ^28^, ^32^ ^]^ have demonstrated that removing the CTCF peak at the upstream boundary of the SOX17 locus reduces interactions between SOX17 and its distant regulatory elements (DRE) while increasing interactions with the upstream boundary near the RGS20 locus. To further assess whether ChromNet can generalize to other perturbation contexts and cell types, we applied the model to predict chromatin interaction changes in murine Th1 cells with a deletion of a CTCF binding site at the Ifng ‐70 kb region.^[^ ^33^ ^]^ As shown in Figure S8 (Supporting Information), ChromNet predictions for wild‐type (WT) and CBS‐70Δ samples revealed clear differences in chromatin contacts around the Ifng locus. These results further demonstrate the robustness of ChromNet in capturing biologically meaningful 3D genome alterations resulting from targeted CTCF loss.

## 3 Conclusion

In this study, we introduce ChromNet, a multi‐task learning framework capable of predicting high‐resolution 3D chromatin structures across diverse cell types, spanning both normal and malignant states. By integrating multi‐cell‐type epigenetic profiles and incorporating noise‐perturbed signals during training, ChromNet demonstrates enhanced generalization across cellular contexts and robust performance against epigenetic signal variations. This design enables the model to capture both conserved chromatin architectural principles and cell‐type‐specific structural reconfigurations.

ChromNet consistently outperforms existing state‐of‐the‐art models in cross‐cell‐type chromatin reconstruction tasks, achieving higher fidelity across multiple common cell lines (e.g., IMR‐90, K562, GM12878, and HCT116). Importantly, when applied to leukemia samples, ChromNet accurately captures disease‐specific chromatin alterations, even in the absence of matched Hi‐C training data. These findings underscore ChromNet's potential as a powerful computational tool for investigating chromatin architecture in clinically relevant contexts, particularly in diseases where experimental Hi‐C profiling is limited by cost, resolution, or sample availability.

Beyond its predictive accuracy, ChromNet opens several avenues for future research. It offers a scalable and cost‐efficient strategy to generate high‐resolution 3D genome maps for large‐scale studies, such as human developmental atlases and cancer epigenomes. Moreover, the framework can be extended to incorporate additional chromatin marks or perturbation datasets (e.g., CRISPRi, CUT&RUN), enabling systematic dissection of causal relationships between epigenomic changes and spatial genome organization. In particular, future extensions of ChromNet could integrate histone post‐translational modification profiles (e.g., H3K27ac, H3K4me3, H3K27me3) to refine the modeling of cell‐type‐specific chromatin architecture and further enhance its biological interpretability. Coupled with downstream analyses, ChromNet could also facilitate the identification of regulatory elements involved in long‐range gene control and support the discovery of structural biomarkers for disease stratification or treatment response.

Furthermore, our findings indicate that the selection of auxiliary cell types during training has a significant impact on generalization performance. Auxiliary cells that share lineage identity, regulatory programs, or epigenomic features—such as chromatin accessibility or CTCF‐binding profiles—with the prediction target provide more transferable context for modeling chromatin structure. This highlights the importance of biologically informed auxiliary selection for improving cross‐cell‐type predictions. Future work may explore systematic approaches to select auxiliary cells based on epigenomic similarity or developmental relevance, particularly in settings involving disease subtypes or rare cell populations.

Ultimately, ChromNet provides a generalizable platform for interrogating the 3D genome, bridging experimental data limitations with computational innovation. Its application may accelerate our understanding of genome regulation in development and disease and inform the design of future therapeutic strategies targeting chromatin architecture.

## 4 Experimental Section

### Hi‐C Data Processing

High‐throughput Hi‐C sequencing data were collected from multiple cell types, including four commonly used cell lines (IMR‐90, GM12878, K562, and HCT116), as well as primary peripheral blood mononuclear cells (PBMC) from healthy donors and acute myeloid leukemia (AML) patient samples. Hi‐C data for the IMR‐90, GM12878, K562, and HCT116 cell lines were obtained from the 4D Nucleome Data Portal. To ensure consistency and minimize bias in Hi‐C data preprocessing, all interaction matrices underwent Knight‐Ruiz (KR) normalization^[^ ^34^ ^]^ using HiCExplorer.^[^ ^35^ ^]^

For downstream analysis, reversible natural logarithm transformations were applied to the processed Hi‐C interaction matrices to maintain compatibility with subsequent computational modeling and visualization.

### Genomic Sequence Processing

The reference genome sequence (hg38) was retrieved from the UCSC Genome Browser and used consistently across all cell types. The original FASTA files contain four nucleotide types, with unknown bases denoted as “N”. To preserve genomic information, the “N” category was retained and encoded as a fifth unknown nucleotide. The one‐hot encoding scheme represents each nucleotide as a five‐channel vector corresponding to “A”, “T”, “C”, “G”, and “N”.

### Epigenomic Data Processing

CTCF ChIP‐seq and ATAC‐seq datasets for all cell types were obtained from the GEO database and the ENCODE Data Portal, with accession numbers provided in Table S1 (Supporting Information). To ensure data quality and consistency across different experimental conditions, systematic preprocessing was performed. A scaling factor was computed based on the ratio of target reads to total reads, allowing for cross‐sample comparability.

The raw sequencing reads were processed using standard alignment and peak‐calling pipelines. To normalize signal intensity, the bamCoverage tool^[^ ^36^ ^]^ was employed to generate bigWig files, applying reads per kilobase per million mapped reads (RPKM) normalization with a bin size of ten.

### PBMC and AML Sample Processing

Hi‐C, CTCF CUT&Tag, and ATAC‐seq data from PBMC and AML patient samples were obtained from the GEO database. The preprocessing pipeline was aligned with the protocols applied to cell line datasets to maintain data consistency. Accession numbers and specific preprocessing parameters are detailed in Table S1 (Supporting Information).

### ChromNet Training Data Processing

ChromNet was trained using reference DNA sequences and epigenomic signals, including CTCF ChIP‐seq and ATAC‐seq data, derived from both IMR‐90 and K562 cell lines. However, the ground truth Hi‐C interaction matrices used for training were exclusively obtained from IMR‐90. This design enables the model to integrate epigenomic signals from multiple cell types while learning chromatin organization patterns specific to IMR‐90.

K562 was chosen as the auxiliary input to IMR‐90 to evaluate generalization across diverse lineages, leveraging its biological difference from IMR‐90. However, comparative results with GM12878 as the auxiliary cell (see Table S7, Supporting Information) show that ChromNet generalizes well with either choice, highlighting that the inclusion of an auxiliary cell is more critical than the specific identity of the auxiliary cell.

In the multi‐task learning framework, the chromatin reconstruction task utilized the Hi‐C interaction matrices from IMR‐90 as labels, whereas the cell‐type classification task employed the corresponding cell type of the input epigenomic signals as labels. Following the methodology of C.Origami, the Hi‐C matrices were initially processed at a 10 kb resolution and subsequently down sampled to 8,192 bp to align with the model's output resolution.

To construct training batches, a 2.1 Mb sliding window with a 500 kb step size was applied across the genome. Genomic regions were partitioned into training, validation, and test sets: chromosomes 1–16 for training, chromosomes 17–19 for validation, and chromosomes 20–22 for testing. Details on training configurations are provided in Table S2 (Supporting Information).

### AML Data Processing

AML‐related data were preprocessed following the same pipeline as the cross‐cell‐type training data. ChromNet was trained on epigenetic features from PBMC_103 and AML_270, including DNA sequences, CTCF CUT&Tag and ATAC‐seq signals. Similar to the standard training pipeline, the Hi‐C interaction matrices used for training were obtained exclusively from PBMC_103. Model performance was evaluated on multiple AML patient samples (e.g., AML_018, AML_472, AML_629) and benchmarked against C.Origami, which was trained only on PBMC_103 epigenomic signals. Training configurations for AML‐related datasets are detailed in Table S3 (Supporting Information).

### Model Architecture

The architecture of ChromNet included a core Chromatin Feature Encoder, followed by two distinct modules: the Chromatin Interaction Reconstructor for chromatin interaction reconstruction and the Cell‐Type Classifier for cell‐type classification.

Chromatin Feature Encoder was responsible for extracting and processing features from the input DNA sequences and epigenetic signals of different cell types. It consists of three key components:

- **Hierarchical Feature Reduction Module**: This module employs multiple layers of convolution and pooling operations to extract critical information from the input signals while preserving important details for chromatin interaction prediction. By hierarchical feature reduction, the model removes redundant information and retains the most biologically significant signals.
- **Resolution‐Adaptable Processing Module**: This module adjusts the resolution of the processed features according to the target resolution for Hi‐C interaction matrix prediction. By dynamically modifying its resolution parameters, the model was capable of generating Hi‐C interaction matrices at different resolutions, ensuring flexibility and accuracy in predicting chromatin interactions based on the required resolution.
- **Contextual Encoding Module**: The processed features were subsequently fed into the Contextual Encoding Module, where a Long Short‐Term Memory (LSTM)^[^ ^37^ ^]^ network was used to capture long‐range dependencies between chromatin segments. The module further applies a dot product operation to compute interactions between chromatin regions, allowing the model to recognize pairwise interactions across the sequence. This enables the model to effectively capture long‐range chromatin interactions, such as topologically associating domains (TADs), enhancing the model's ability to incorporate global chromatin context in its predictions.

The Chromatin Feature Encoder extracts features for both chromatin interaction reconstruction and cell‐type classification tasks. In the cell‐type classification task, features from the target cell type, along with those from other cell types, were fed into the Cell‐Type Classifier. This classifier learns to distinguish different cell types by capturing epigenetic and chromatin feature patterns, thereby encouraging the encoder to focus on variations in epigenetic signals across different cell types at the same genomic regions. This process enables the encoder to learn cell‐type‐specific variations while preserving shared features across cell types, enhancing its generalization ability and ultimately improving chromatin interaction prediction accuracy. During chromatin interaction reconstruction, only the features of the target cell type are used to ensure that the predicted chromatin structure aligns with the epigenetic state of that cell type.

The Chromatin Interaction Reconstructor, built on a U‐Net^[^ ^38^ ^]^ style architecture, reconstructs chromatin interaction matrices from high‐dimensional features derived from the Chromatin Feature Encoder. Using dilated convolutions in the encoding path, it captures long‐range chromatin interactions across multiple levels. The symmetrical decoding path progressively restores spatial resolution, while residual connections facilitate multi‐scale feature integration. Through successive convolutional and decoding layers, the module generates the Hi‐C interaction matrix, effectively capturing both global chromatin architecture and local interactions. Global chromatin architecture, such as topologically associating domains (TADs), was captured through the model's ability to aggregate information across long genomic distances, while local interactions are refined at higher resolutions in the decoding path. This enables the model to effectively capture hierarchical chromatin organization.

Cell‐Type Classifier generates predictions of cell types based on the features provided by the Chromatin Feature Encoder. By processing the input signals through convolutional layers and fully connected layers, this classifier outputs the predicted cell types. The incorporation of the cell‐type classification task enhances the model's ability to capture differences between various cell types, thereby improving its generalization capabilities.

### Feature Integration and Cell‐Type Generalization

Predicting chromatin structure across different cell types requires encoding both conserved and cell‐type‐specific chromatin features. To enhance ChromNet's generalization, a novel approach was introduced by perturbing the epigenetic signals of the target cell type with Gaussian noise, simulating the epigenetic differences between cell types. Although the DNA sequence remains the same, differences in epigenetic signals like CTCF and ATAC‐seq can significantly influence chromatin interactions. The noise perturbation helps the model learn how these signals affect chromatin structure.

In the Chromatin Feature Encoder, the model integrates the noise‐perturbed features of the target cell type *F*_*target*_ with the features from other cell types *F*_*i*_(*i* ≠ *target*), allowing it to capture both conserved chromatin interaction patterns and cell‐type‐specific differences. This integration can be expressed as:

(1) $$F_{encoded}=E\left(F_{target}+\varepsilon ,F_{1},F_{2},\text{…},F_{N}\right)$$

where *E*(·) denotes the feature extraction function of the Chromatin Feature Encoder, and ϵ is noise sampled from a Gaussian distribution $N(0,\sigma^{2})$. By adding noise to the epigenetic signals of the target cell type, the model becomes more adept at identifying subtle regulatory impacts and complex interactions between epigenetic features and chromatin structure. This method, in conjunction with integrating signals from other cell types, enables the model to generalize more effectively and predict chromatin structure in previously unseen cell types.

### Multi‐Task Learning Strategy

ChromNet adopted a multi‐task learning framework, simultaneously performing chromatin interaction prediction and cell‐type classification. Specifically, the model outputs included both reconstructed chromatin interaction matrices and predicted cell types, and it optimizes the following joint loss function:

(2) $$L_{total}=L_{recon}+L_{class}$$

where *L*_*recon*_ represents the loss for chromatin interaction prediction, *L*_*class*_ represents the loss for cell‐type classification.

### Training Strategy

During training, ChromNet employed the Adam optimizer with a learning rate warm‐up and cosine annealing to ensure stability and convergence. Before noise injection, all epigenetic signals were log‐transformed to reduce scale disparities across modalities (e.g., CTCF, ATAC‐seq). During training, both the unperturbed epigenetic signals and their noisy versions were simultaneously input, where fixed Gaussian noise (mean = 0, standard deviation = 0.1) was uniformly added across epigenetic modalities. To match the noisy inputs, Gaussian noise (mean = 0, standard deviation = 0.1) was also added to each valid contact value in the corresponding ground truth Hi‐C matrices, which were then paired with the noisy epigenetic signals for training. This strategy of using both noisy and clean data encourages the model to retain essential signal features while enhancing its robustness to potential fluctuations and experimental noise in the input signals and Hi‐C data. The chosen standard deviation of 0.1 balances introducing sufficient variation with preserving the key data characteristics. During validation and testing, the model uses only noise‐free epigenetic signals, and predictions were directly compared to the experimental noise‐free Hi‐C data. All training was performed on an NVIDIA V100 GPU with 32GB of RAM.

During inference (de novo prediction on unseen cell types), only the reference DNA sequence and the epigenetic signals (CTCF and ATAC‐seq) from the new cell type are used. No information from other cell types was included.

### Model Parameters

ChromNet consists of three core modules: the Chromatin Feature Encoder, Chromatin Interaction Reconstructor, and Cell‐Type Classifier, designed to optimize chromatin structure prediction.

The Chromatin Feature Encoder includes three components. The hierarchical feature reduction module comprises three convolutional layers, with input channels set to seven. The first layer outputs 16 channels, the second outputs 32, and the third outputs 64, all with a kernel size of 3 and a stride of 1. The resolution‐adaptable processing module consists of two Top‐*k* pooling layers, with a patch length of 10 and a Top‐*k* value of 10 to retain key features. The contextual encoding module employed an LSTM with a sequence dimension of 128 and a hidden dimension of 256 to capture long‐range dependencies.

The Chromatin Interaction Reconstructor follows a U‐Net‐style decoder architecture, integrating global and local features through an encoding‐decoding framework. The input feature map first passes through an initial convolutional layer, adjusting the channel size from 128 to 256, with a kernel size of 3 and a stride of 1. It then undergoes five groups of residual blocks for deep feature extraction. The first group maintains 256 channels, while the subsequent four groups reduce channels to 128, 64, 32, and 16, respectively, each containing five residual blocks with dilated convolutions to enhance long‐range dependency modeling. In the encoding path, convolutional layers progressively compress feature maps, reducing channel dimensions, while the decoding path restores spatial resolution through up sampling, ultimately outputting 256 channels. The final convolutional layer produces a single‐channel Hi‐C interaction matrix, ensuring precise chromatin interaction reconstruction.

The Cell‐Type Classifier processes features using convolutional and fully connected layers to generate cell‐type predictions. The input consisted of 256 channels, which were reduced to 128 and then 64 through two convolutional layers. The flattened features were passed through fully connected layers, reducing to 256 dimensions before the final cell‐type classification output. This auxiliary task improves the model's ability to distinguish between cell types, enhancing generalization.

### Insulation Score Correlation

The insulation score quantifies the degree of chromatin domain insulation, where lower values indicate stronger domain boundaries due to reduced interactions across adjacent regions. In this study, the insulation score was computed using the HiCplotter tool,^[^ ^39^ ^]^ applying a sliding‐window approach to integrate interaction frequencies within a predefined genomic range. The accuracy of chromatin domain prediction was evaluated by computing the Pearson and Spearman correlation coefficients between the predicted and experimentally derived insulation scores.

### Distance‐Stratified Correlation

To assess the model's predictive performance across different genomic scales, distance‐stratified correlation analysis was employed. This method quantified prediction accuracy across various genomic distances by computing correlations along different offset diagonals of the Hi‐C interaction matrix. Specifically, for a given Hi‐C interaction matrix, chromatin interaction intensities were stratified based on genomic distance. For each offset distance d, all interaction values corresponding to that distance were extracted, and the Pearson and Spearman correlation coefficients between the predicted and experimental matrices were computed.

### Dynamic Time Warping (DTW) Analysis

To evaluate the model's ability to capture chromatin topology dynamics in a disease context, Dynamic Time Warping (DTW) was adopted to assess the similarity between predicted and experimentally observed insulation score changes during the PBMC‐to‐AML transition.

Let $S^{\mathrm{PBMC}}\in R^{n}$ and $S^{\mathrm{AML}}\in R^{n}$ denote the insulation scores across genomic bins for PBMC and AML, respectively. Each vector was standardized via z‐score normalization:

(3) $${\tilde{S}}^{\mathrm{PBMC}}=\frac{S^{\mathrm{PBMC}}-\mu_{\mathrm{PBMC}}}{\sigma_{\mathrm{PBMC}}}$$

(4) $$\;{\tilde{S}}^{\mathrm{AML}}=\frac{S^{\mathrm{AML}}-\mu_{\mathrm{AML}}}{\sigma_{\mathrm{AML}}}$$

where μ and σ denote the mean and standard deviation of each insulation score vector.

Next, the differential insulation profile was computed:

(5) $$\Delta S={\tilde{S}}^{\mathrm{PBMC}}-{\tilde{S}}^{\mathrm{AML}}$$

and obtain its predicted counterpart $\hat{\Delta S}$ following the same procedure.

The DTW distance between Δ*S* and $\hat{\Delta S}$ is then computed as:

(6) $$D_{\mathrm{DTW}}\left(\Delta S,\hat{\Delta S}\right)=\min_{W}\sum_{k=1}^{L}{\left(\Delta S_{i_{k}}-{\hat{\Delta S}}_{j_{k}}\right)}^{2}$$

where *W* = {(*i*_1_, *j*_1_), …, (*i*_*L*_, *j*_*L*_)} is a warping path that satisfies the standard monotonicity and continuity constraints.

## Conflict of Interest

The authors declare no conflict of interest.

## Author Contributions

J.X.W. conceived and designed this project. B.W. conceived, designed, and implemented the ChromNet. S.K.W. and L.Q.D. provided valuable guidance drawing on their expertise in biology. J.X.W. and B.W. wrote the paper. S.K.W, H.D.L., and Y.H.L. revised and proofread the manuscript. All authors have read and approved the final version of this paper.

## Supporting information

Supporting Information

## Data Availability

Data sharing is not applicable to this article as no new data were created or analyzed in this study.
