# Supplementary material for: ChromNet: A Multi‐Task Learning Framework for Cross‐Cell Type Prediction of 3D Chromatin Interactions Using Epigenetic Signals
Source: Adv Sci (Weinh). 2025 Oct 30;13(1):e08110. doi: 10.1002/advs.202508110 (PMC12767100; doi:10.1002/advs.202508110)
Supplement: Supplementary file 1 — Supporting Information [file ADVS-13-e08110-s001.pdf]

Supplementary Information for

ChromNet: A Multi-task Learning Framework for Cross-cell Type Prediction of 3D Chromatin Interactions Using Epigenetic Signals

Supplementary Figures

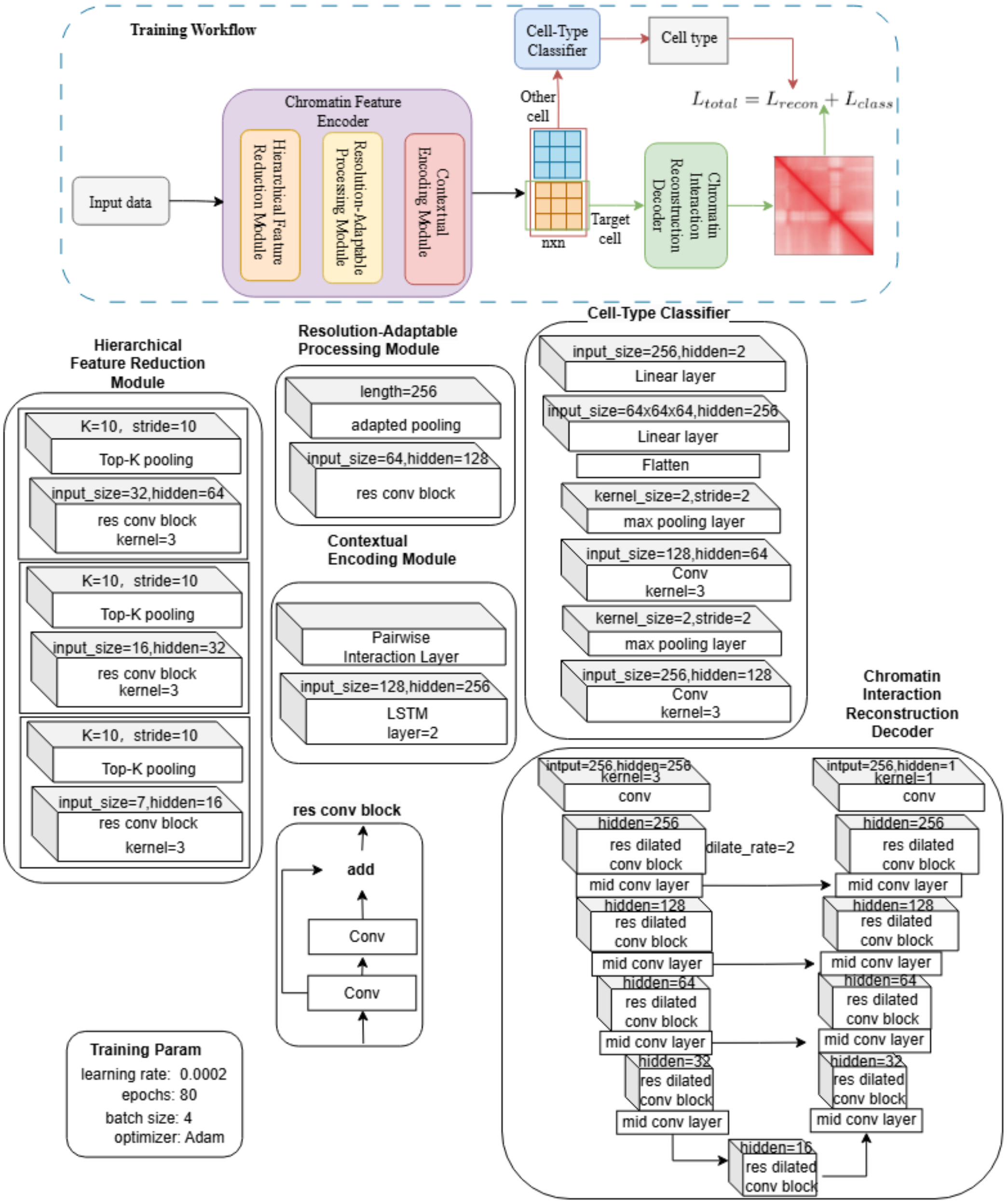

Fig. S1. ChromNet Architecture and Parameter Configuration. The detailed architecture of the ChromNet model, highlighting key components such as the Chromatin Feature Encoder, Chromatin Interaction Reconstructor, and Cell-Type Classifier. The parameters corresponding to each module, including kernel sizes, strides, and output dimensions.

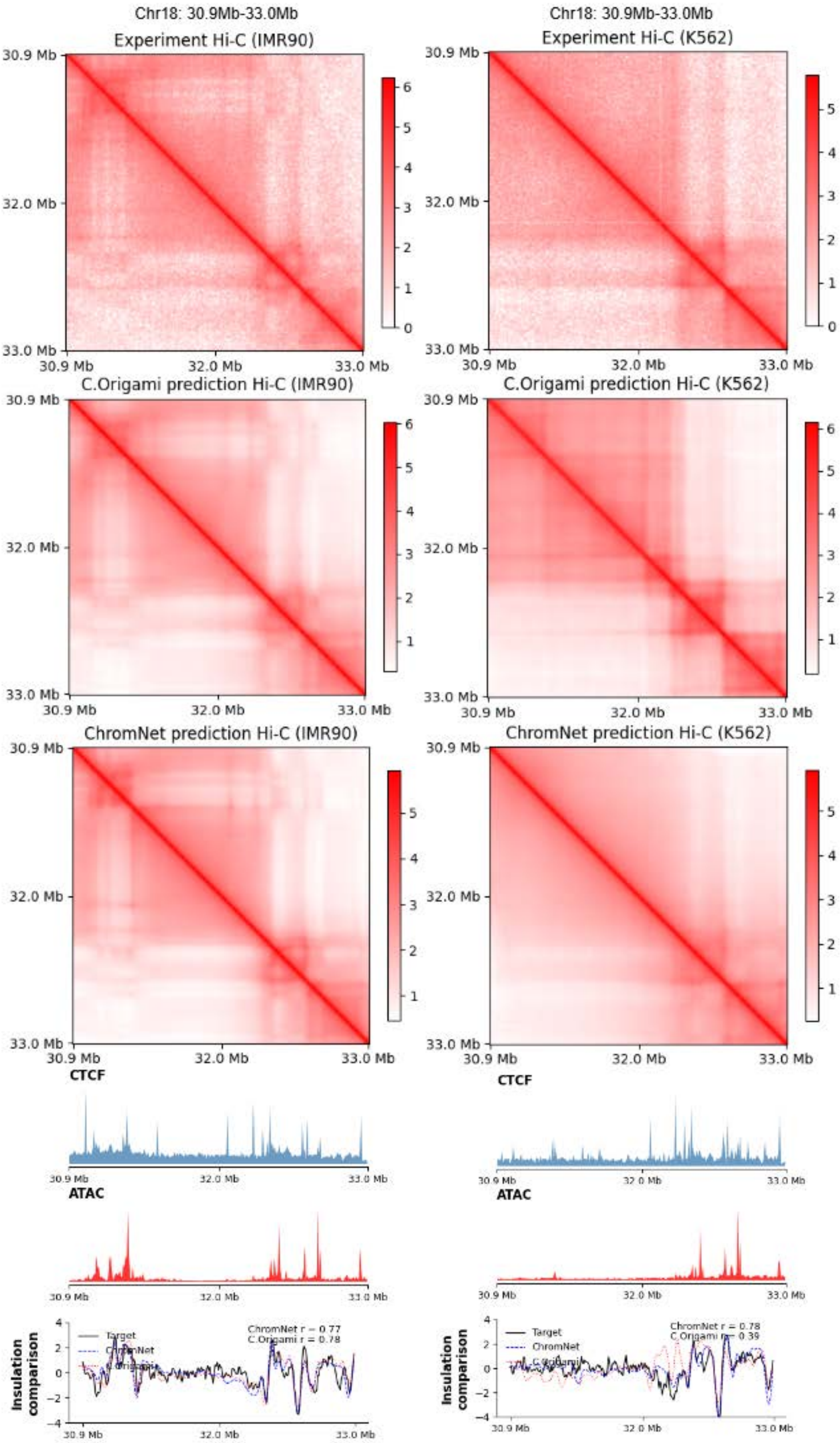

Fig. S2. Visualization of chromatin interaction predictions for K562 across cell types. Hi-C contact matrices and associated chromatin features are shown for a representative region from two cell types: IMR-90 (left) and K562 (right). From top to bottom, each panel displays the experimental Hi-C matrix (Experiment Hi-C), C.Origami prediction, and ChromNet prediction, all at 8,192 bp resolution. Below each matrix panel, corresponding CTCF and ATAC-seq signals are plotted for the same genomic interval. The bottom panel presents the normalized insulation scores derived from each Hi-C matrix (Experiment, ChromNet, and C.Origami). Pearson correlation coefficients between predicted and experimental insulation scores are reported.

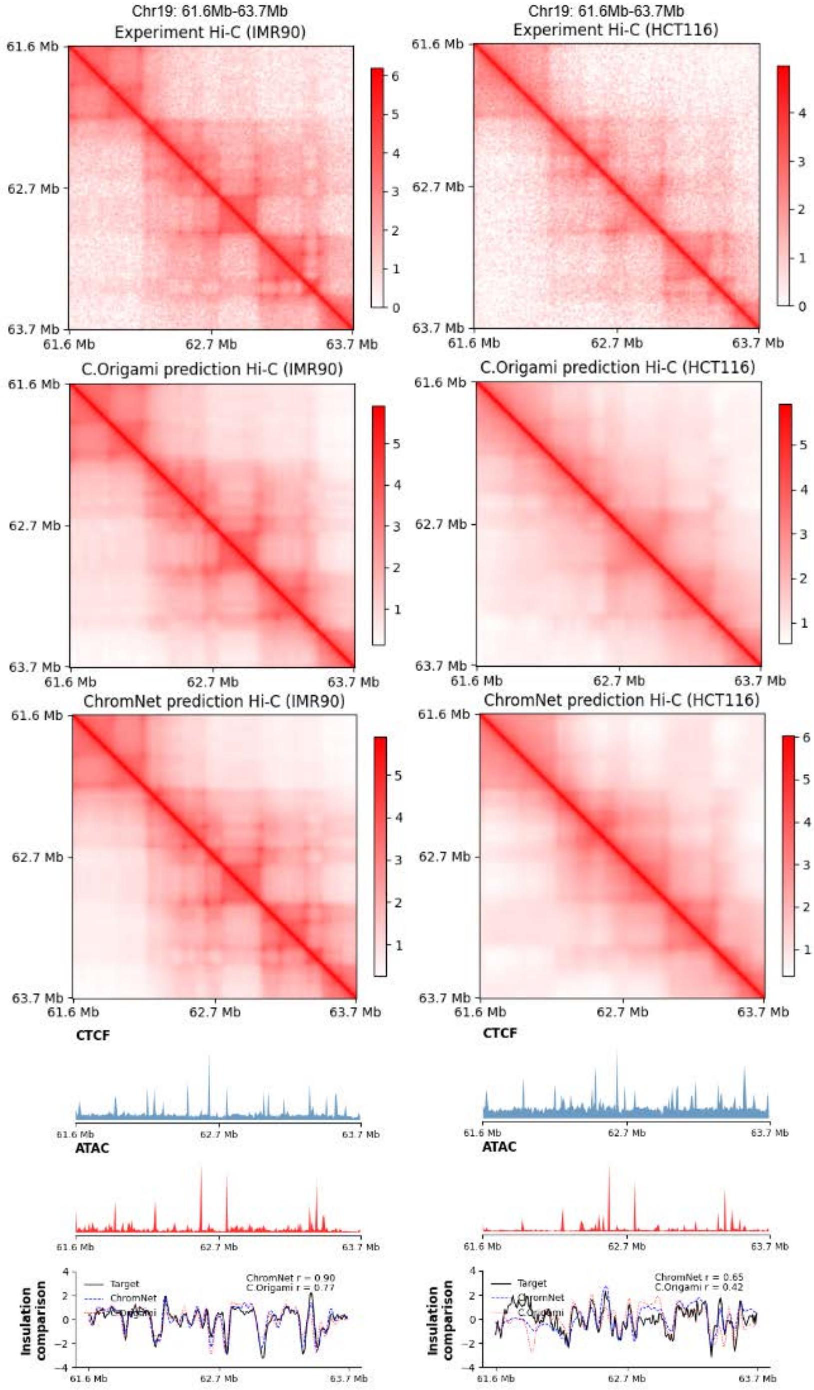

Fig. S3. Visualization of chromatin interaction predictions for HCT116 across cell types. Hi-C contact matrices and associated chromatin features are shown for a representative region from two cell types: IMR-90 (left) and HCT116 (right). From top to bottom, each panel displays the experimental Hi-C matrix (Experiment Hi-C), C.Origami prediction, and ChromNet prediction, all at 8,192 bp resolution. Below each matrix panel, corresponding CTCF and ATAC-seq signals are plotted for the same genomic interval. The bottom panel presents the normalized insulation scores derived from each Hi-C matrix (Experiment, ChromNet, and C.Origami). Pearson correlation coefficients between predicted and experimental insulation scores are reported.

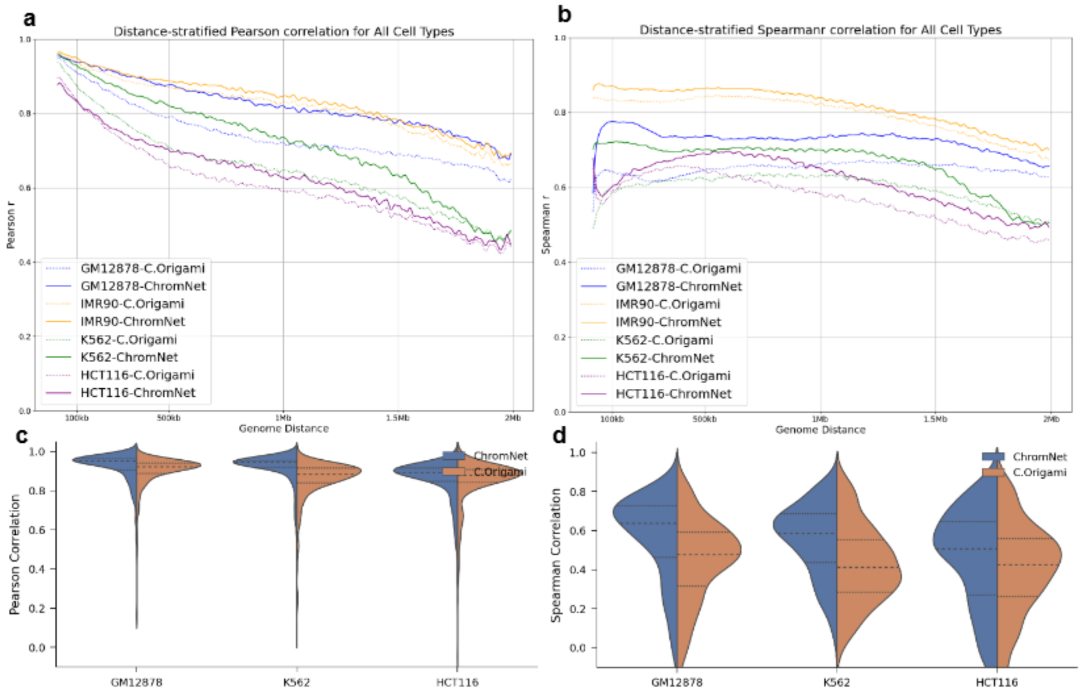

Fig. S4. Performance comparison of ChromNet and C.Origami in insulation score and distance-stratified correlations across different cell types. a, b Distance-stratified Pearson and Spearman correlation curves for all cell types, illustrating the predictive performance of ChromNet and C.Origami at varying genomic distances. **c, d** Violin plots showing the distribution of insulation score correlations (Pearson and Spearman, respectively) for ChromNet (blue) and C.Origami (orange) across different cell types.

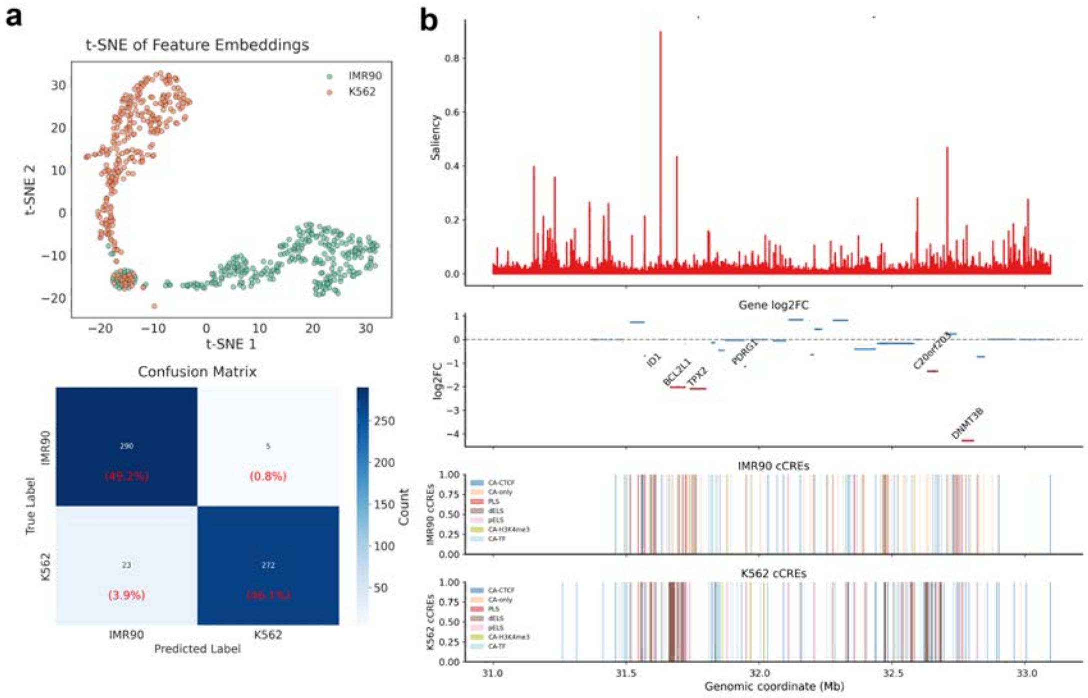

Fig. S5. Analysis of ChromNet auxiliary classifier feature representations and saliency in IMR-90 and K562 test sets. a t-SNE visualization of feature embeddings extracted from the penultimate layer of the auxiliary classifier for test sets, with IMR-90 (green) and K562 (orange) forming distinct clusters, and accompanying confusion matrix showing the classification performance. **b** Detailed analysis of the chr20:31–33 Mb region selected as a representative example; **Top panel** shows the saliency scores along genomic coordinates, computed using Integrated Gradients (IG). **Second panel** displays gene-level log2 fold changes (IMR-90 vs. K562) for differentially expressed genes within the region, with names of key upregulated genes labeled (|log2FC|>1). **Third and fourth panels** illustrate IMR90 and K562 candidate cis-Regulatory Elements(cCRE) annotations[1], respectively, with colored vertical lines representing different classes of cCREs, including Proximal Enhancer-like Signature (pELS), Distal Enhancer-like Signature (dELS), Promoter-like Signature (PLS), Chromatin Accessible with CTCF (CA-CTCF), Chromatin Accessible with H3K4me3 (CA-H3K4me3), Chromatin Accessible with Transcription Factor binding (CA-TF), and Chromatin Accessible Only (CA-only).

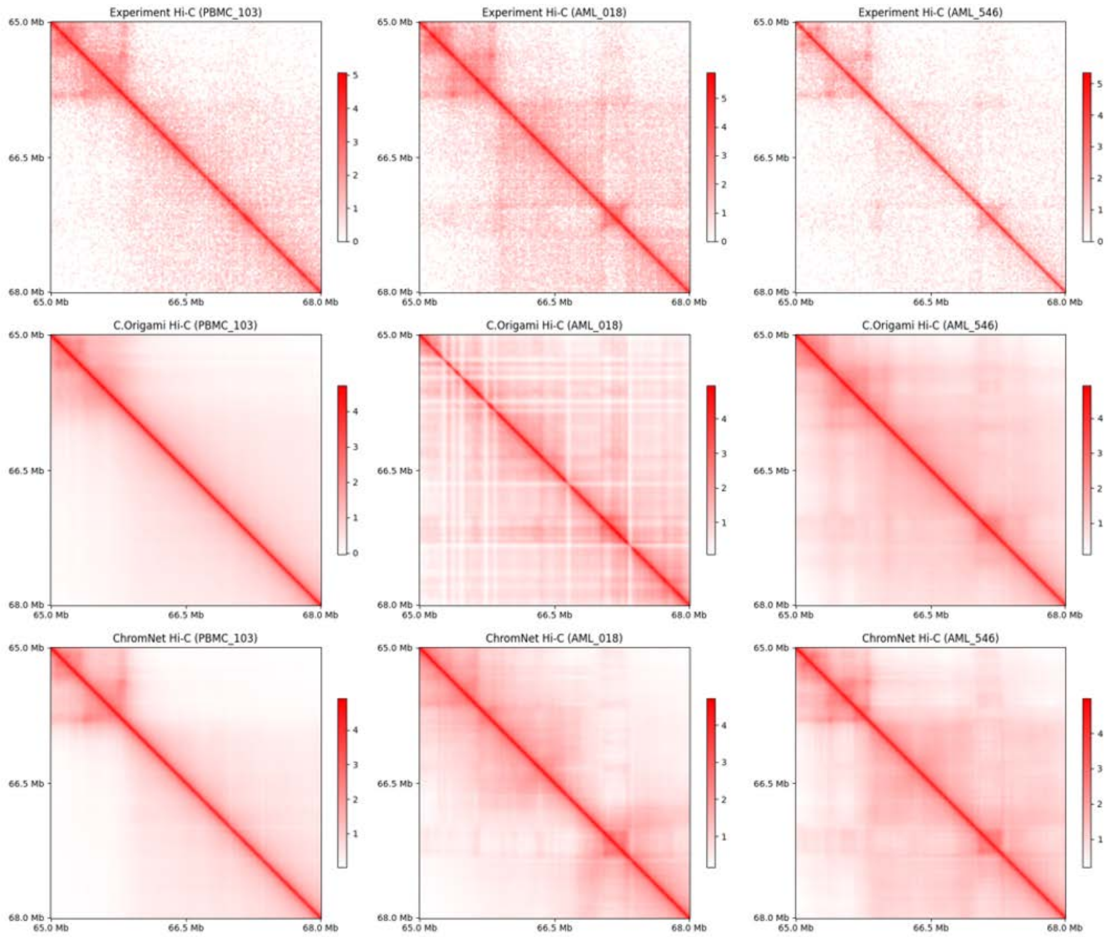

Fig. S6. Visualization of Chromatin Interaction Predictions for PBMC_103 and AML Samples. Each row contains three panels representing chromatin interaction maps for PBMC_103, AML_018, and AML_546. The top row presents the experimental Hi-C data for each sample. The middle row shows the predicted interaction maps generated by C.Origami, while the bottom row illustrates the predictions from ChromNet.

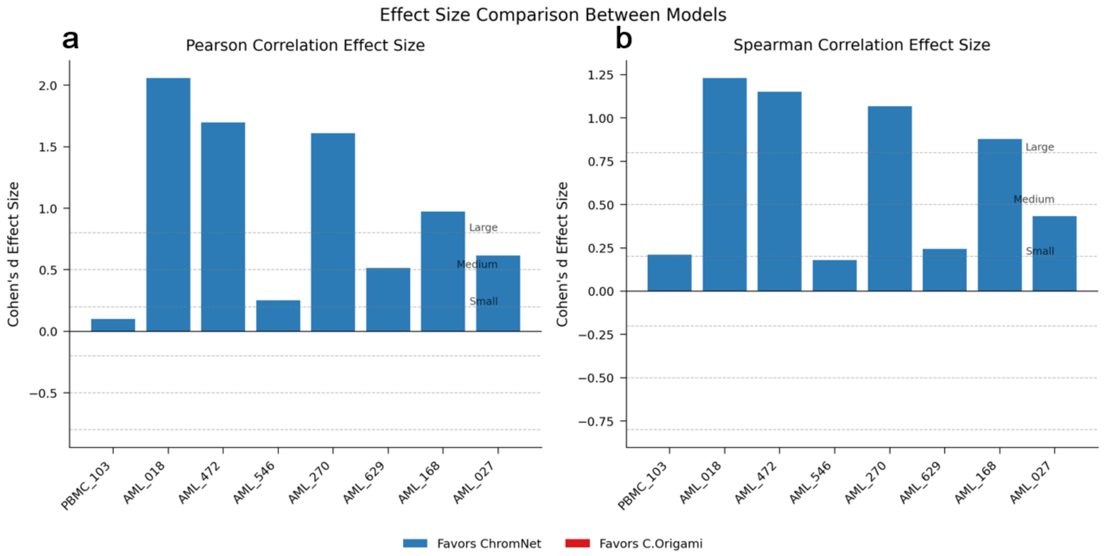

Fig. S7. Effect Size Comparison Between ChromNet and C.Origami Based on Insulation Score Correlations. a Cohen's d effect size for insulation score Pearson correlation between ChromNet and C.Origami across different cell types. A positive effect size indicates that ChromNet outperforms C.Origami. Dashed horizontal lines represent standard effect size thresholds: small (0.2), medium (0.5), and large (0.8). **b** Cohen's d effect size for Spearman correlation, following the same convention as **a**. ChromNet consistently shows larger effect sizes across multiple cell types, indicating superior performance. The color legend at the bottom indicates which method is favored.

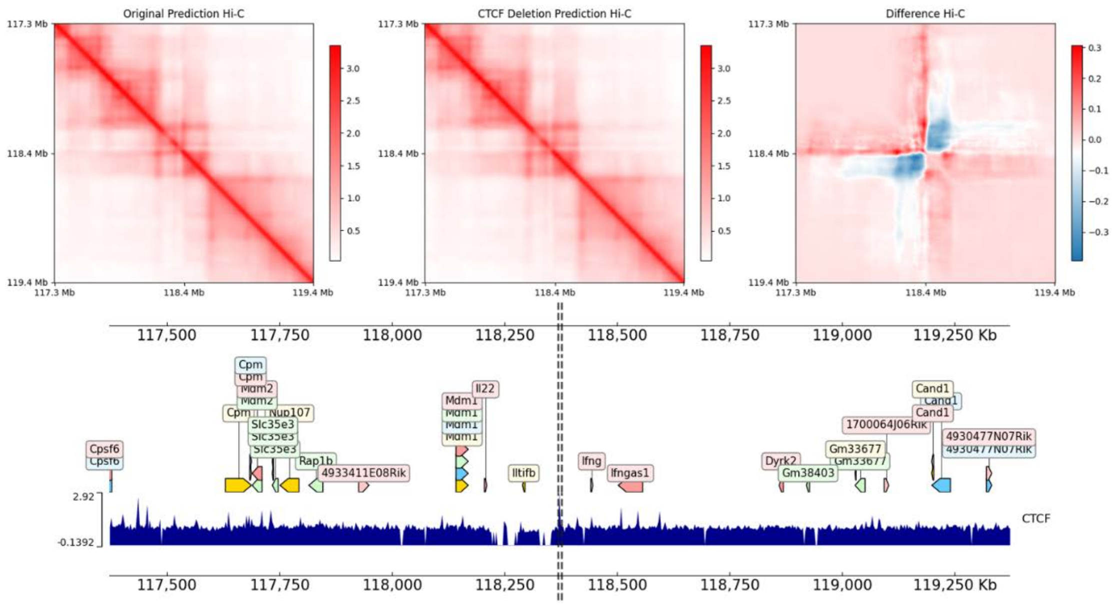

Fig. S8. Impact of CTCF Binding Site Deletion on Chromatin Interactions in Th1 Cells. The effect of CTCF binding site deletion on the 3D chromatin structure in Th1 cells, focusing on the *Ifng -70* kb region. The top row presents Hi-C contact maps: the left panel shows the original Hi-C prediction for wild-type (WT) cells, the middle panel depicts the Hi-C prediction for *CBS-70Δ* cells (CTCF binding site deletion), and the right panel displays the difference matrix, highlighting chromatin interaction changes due to CTCF deletion (red for decreased interactions and blue for increased interactions). The bottom panel provides genomic context, showing the gene annotations and CTCF binding signals at the corresponding genomic region.

Supplementary Tables

Table S1. Accession numbers of epigenetic and Hi-C datasets used in this study. Lists ENCODE or GEO accession IDs for CTCF, ATAC-seq, and Hi-C data across all cell types included in ChromNet and C.Origami experiments, ensuring reproducibility and transparency of data sources.

**Cell type**	**CTCF**	**ATAC-seq**	**Hi-C data**
IMR-90	ENCSR000EFI	ENCSR200OML	4DNES1ZEJNRU
GM12878	ENCSR000DZN	ENCSR637XSC	4DNES3JX38V5
K562	ENCSR000EGM	ENCSR868FGK	4DNESI7DEJTM
HCT116	ENCSR240PRQ	ENCSR872WGW	4DNESNSTBMBY
PBMC and AMLs	GSE152132	GSE152134	GSE152135

Table S2. Cross-cell-type training configuration for ChromNet and C.Origami. Details the input modalities, supervision labels, and chromosome-level dataset partitioning used for training and evaluation in common cell line experiments (IMR-90, K562, GM12878, HCT116).

**Description**	**ChromNet**	**C.Origami**
Input Data	IMR-90, K562 (DNA sequences, CTCF, ATAC)	IMR-90 (DNA sequences, CTCF, ATAC)
Chromatin Reconstruction Labels	Hi-C matrix (IMR-90)	Hi-C matrix (IMR-90)
Dataset Partitioning	Train: Chr 1-16, Val: Chr 17-19, Test: Chr 20-22	Train: Chr 1-16, Val: Chr 17-19, Test: Chr 20-22

Table S3. AML training configuration for ChromNet and C.Origami. Describes the input data composition, chromatin reconstruction supervision, and train-validation-test chromosome splits for experiments involving PBMC_103 and AML patient samples.

**Description**	**ChromNet**	**C.Origami**
Input Data	PBMC_103, AML_270 (DNA sequences, CTCF, ATAC)	PBMC_103 (DNA sequences, CTCF, ATAC)
Chromatin Reconstruction Labels	Hi-C matrix (PBMC_103)	Hi-C matrix (PBMC_103)
Dataset Partitioning	Train: Chr 1-16, Val: Chr 17-19, Test: Chr 20-22	Train: Chr 1-16, Val: Chr 17-19, Test: Chr 20-22

Table S4. Performance comparison of ChromNet and C.Origami across various cell types. Models were trained on IMR-90 Hi-C: ChromNet incorporated auxiliary epigenetic signals from K562, whereas C.Origami was trained using only IMR-90's own epigenetic features, following its original configuration. Metrics include insulation score Pearson/Spearman correlations and distance-stratified correlations for K562, GM12878, and HCT116. Higher values indicate better predictive accuracy of chromatin structure.

Methods	Insul. P.Corr.	Insul. S.Corr.	Dist. P.Corr.	Dist. S.Corr.
		**K562**		
C.Origami	0.8569	0.4294	0.6963	0.6788
ChromNet	**0.9092**	**0.5519**	**0.7534**	**0.7333**
		**GM12878**		
C.Origami	0.8953	0.4463	0.7850	0.7198
ChromNet	**0.9147**	**0.5698**	**0.8492**	**0.7723**
		**HCT116**		
C.Origami	0.8393	0.4086	0.6366	0.6328
ChromNet	**0.8513**	**0.4522**	**0.6477**	**0.6675**

*Note: Bold values indicate the best performance for each metric.

Table S5. Ablation experiment: performance metrics of ChromNet variants across various cell types. All models were trained on IMR-90 Hi-C. "ChromNet_base" used only IMR-90 epigenetic data without auxiliary cell or noise; "ChromNet_noise" added Gaussian noise to IMR-90 CTCF and ATAC-seq; "ChromNet" used K562 as the auxiliary cell. Results include insulation and distance-stratified correlations, reflecting model robustness and generalization.

Methods	Insul. P.Corr.	Insul. S.Corr.	Dist. P.Corr.	Dist. S.Corr.
		**IMR-90**		
ChromNet	**0.9356**	**0.7484**	0.8441	0.8465
ChromNet_base	0.9257	0.7113	0.8374	0.8371
ChromNet_noise	0.9290	0.7443	**0.8465**	**0.8472**
		**K562**		
ChromNet	**0.9092**	**0.5519**	**0.7534**	**0.7333**
ChromNet_base	0.8697	0.4066	0.7131	0.6740
ChromNet_noise	0.8298	0.4287	0.7257	0.6955
		**GM12878**		
ChromNet	**0.9147**	**0.5698**	**0.8492**	0.7723
ChromNet_base	0.8380	0.4122	0.7929	0.7148
ChromNet_noise	0.8853	0.5135	0.8474	**0.7767**
		**HCT116**		
ChromNet	**0.8513**	**0.4522**	**0.6477**	**0.6675**
ChromNet_base	0.8238	0.3609	0.6437	0.6466
ChromNet_noise	0.8329	0.4084	0.6214	0.6341

*Note: Bold values indicate the best performance for each metric.

Table S6. Performance comparison of ChromNet with architectural variants in cross-cell-type prediction. The three additional model variants: "ChromNet_Dec", "ChromNet_Tran", and "ChromNet_Enc", each designed to replace specific modules of ChromNet with corresponding components from C.Origami. "ChromNet_Dec" replaces ChromNet's decoder (U-Net structure) with C.Origami's decoder. "ChromNet_Tran" substitutes the LSTM-based sequence modeling module with the transformer-based architecture from C.Origami. "ChromNet_Enc" replaces the Hierarchical Feature Reduction Module and Resolution-Adaptable Processing Module with C.Origami's encoder structure.

Methods	Insul. P.Corr.	Insul. S.Corr.	Dist. P.Corr.	Dist. S.Corr.
		**IMR90**		
ChromNet	**0.9356**	**0.7484**	**0.8441**	**0.8465**
ChromNet_Dec	0.9336	0.7333	0.8423	0.8443
ChromNet_Tran	0.9102	0.6606	0.8326	0.8353
ChromNet_Enc	0.9222	0.7156	0.8215	0.8222
		**GM12878**		
ChromNet	**0.9147**	**0.5698**	**0.8492**	**0.7723**
ChromNet_Dec	0.9053	0.5592	0.8427	0.7523
ChromNet_Tran	0.8619	0.4453	0.7222	0.7117
ChromNet_Enc	0.9038	0.5221	0.8261	0.7455
		**K562**		
ChromNet	**0.9092**	**0.5519**	**0.7534**	**0.7333**
ChromNet_Dec	0.8937	0.5172	0.7514	0.7302
ChromNet_Tran	0.7756	0.3071	0.4532	0.4418
ChromNet_Enc	0.8456	0.3889	0.6093	0.6307
		**HCT116**		
ChromNet	**0.8513**	**0.4522**	**0.6477**	**0.6675**
ChromNet_Dec	0.8438	0.4482	0.6436	0.6615
ChromNet_Tran	0.8414	0.3986	0.6412	0.6506
ChromNet_Enc	0.8507	0.4363	0.6044	0.6250

*Note: Bold values indicate the best performance for each metric.

Table S7. Performance comparison of ChromNet models trained on IMR-90 Hi-C with different auxiliary cell types. “ChromNet” indicates models trained with K562 as the auxiliary cell, while “ChromNet(GM12878)” uses GM12878 as the auxiliary cell. “ChromNet(NoAux)” denotes training without any auxiliary cell input. Results show insulation score Pearson/Spearman correlations and distance-stratified correlations for four target cell types (IMR-90, K562, GM12878, HCT116). Higher values indicate better prediction accuracy.

Methods	Insul. P.Corr.	Insul. S.Corr.	Dist. P.Corr.	Dist. S.Corr.
		**IMR-90**		
ChromNet(NoAux)	0.9290	0.7443	**0.8465**	**0.8472**
ChromNet	**0.9356**	0.7484	0.8441	0.8465
ChromNet(GM12878)	0.9308	**0.7498**	0.8456	0.8456
		**K562**		
ChromNet(NoAux)	0.8298	0.4287	0.7257	0.6955
ChromNet	**0.9092**	**0.5519**	**0.7534**	**0.7333**
ChromNet(GM12878)	0.8816	0.4826	0.7335	0.7154
		**GM12878**		
ChromNet(NoAux)	0.8853	0.5135	0.8474	0.7767
ChromNet	0.9147	0.5698	0.8492	0.7723
ChromNet(GM12878)	**0.9172**	**0.5801**	**0.8669**	**0.8037**
		**HCT116**		
ChromNet(NoAux)	0.8329	0.4084	0.6214	0.6341
ChromNet	0.8513	0.4522	**0.6477**	**0.6675**
ChromNet(GM12878)	**0.8520**	**0.4690**	0.6469	0.6594

*Note: Bold values indicate the best performance for each metric.

Table S8. Effect of noise level on ChromNet cross-cell-type prediction performance. Models were trained on IMR-90 Hi-C with auxiliary input from K562. Gaussian noise was applied to IMR-90 epigenetic signals at different standard deviations (SD=0.1, 0.4, 0.7). The table reports insulation and distance-stratified correlations for IMR-90, GM12878, K562, and HCT116, assessing how noise magnitude influences generalization.

SD_param	Insul. P.Corr.	Insul. S.Corr.	Dist. P.Corr.	Dist. S.Corr.
		**IMR90**		
ChromNet (0.1)	**0.9356**	**0.7484**	0.8441	**0.8465**
ChromNet (0.4)	0.9307	0.7361	**0.8443**	0.8457
ChromNet (0.7)	0.9302	0.7290	0.8392	0.8404
		**GM12878**		
ChromNet (0.1)	**0.9147**	**0.5698**	0.8492	0.7723
ChromNet (0.4)	0.9044	0.5321	**0.8571**	**0.7854**
ChromNet (0.7)	0.8695	0.4731	0.8539	0.7828
		**K562**		
ChromNet (0.1)	**0.9092**	**0.5519**	**0.7534**	**0.7333**
ChromNet (0.4)	0.8971	0.5015	0.7475	0.7301
ChromNet (0.7)	0.8218	0.3937	0.7508	0.7219
		**HCT116**		
ChromNet (0.1)	**0.8513**	**0.4522**	**0.6477**	**0.6675**
ChromNet (0.4)	0.8420	0.4472	0.6456	0.6641
ChromNet (0.7)	0.8487	0.4481	0.6413	0.6579

*Note: Bold values indicate the best performance for each metric.

Table S9. Performance comparison of ChromNet with fixed vs. dynamically scaled noise strategies across multiple cell types. ChromNet was evaluated using three dynamic noise strategies: ChromNet-CTCF-Fixed (CTCF noise SD fixed at 0.1, ATAC noise SD scaled by the genome-wide mean ratio), ChromNet-ATAC-Fixed (ATAC noise SD fixed at 0.1, CTCF noise SD scaled by the genome-wide mean ratio), and ChromNet-Joint-Scaled (total noise SD=0.2 distributed proportionally based on genome-wide means of CTCF and ATAC). The mean ratio was calculated as (feature_mean / reference_mean) × 0.1, where reference_mean corresponds to the fixed feature (CTCF or ATAC) in each strategy. Performance metrics include insulation score Pearson/Spearman correlations and distance-stratified correlations for IMR-90, K562, GM12878, and HCT116, comparing these variants against the original ChromNet with fixed SD=0.1 noise.

Methods	Insul. P.Corr.	Insul. S.Corr.	Dist. P.Corr.	Dist. S.Corr.
**IMR-90**				
ChromNet	**0.9356**	**0.7484**	**0.8441**	**0.8465**
ChromNet-CTCF-Fixed	0.9308	0.7460	0.8424	0.8453
ChromNet-ATAC-Fixed	0.9307	0.7432	0.8439	0.8462
ChromNet-Joint-Scaled	0.9318	0.7476	0.8415	0.8440
**K562**				
ChromNet	**0.9092**	**0.5519**	0.7534	0.7333
ChromNet-CTCF-Fixed	0.9025	0.5429	0.7603	0.7389
ChromNet-ATAC-Fixed	0.9051	0.5437	**0.7617**	**0.7426**
ChromNet-Joint-Scaled	0.9040	0.5504	0.7538	0.7345
**GM12878**				
ChromNet	**0.9147**	**0.5698**	0.8492	0.7723
ChromNet-CTCF-Fixed	0.9054	0.5514	0.8546	0.7805
ChromNet-ATAC-Fixed	0.8993	0.5486	**0.8580**	**0.7861**
ChromNet-Joint-Scaled	0.9074	0.5587	0.8526	0.7783
**HCT116**				
ChromNet	**0.8513**	**0.4522**	0.6477	0.6675
ChromNet-CTCF-Fixed	0.8504	0.4512	0.6401	0.6549
ChromNet-ATAC-Fixed	0.8505	0.4517	**0.6535**	**0.6717**
ChromNet-Joint-Scaled	0.8512	0.4516	0.6482	0.6678

*Note: Bold values indicate the best performance for each metric.

Table S10. Performance impact of different auxiliary epigenetic features in ChromNet training. Models were trained on IMR-90 Hi-C with different feature subsets: "ChromNet" with full CTCF+ATAC, "ChromNet_onlyCTCF" with only CTCF, and "ChromNet_onlyATAC" with only ATAC-seq. Metrics across IMR-90, K562, GM12878, and HCT116 show the contribution of each feature type to chromatin structure prediction.

Methods	Insul. P.Corr.	Insul. S.Corr.	Dist. P.Corr.	Dist. S.Corr.
**IMR-90**				
ChromNet	**0.9356**	**0.7484**	**0.8441**	**0.8465**
ChromNet_onlyCTCF	0.9208	0.7108	0.8289	0.8278
ChromNet_onlyATAC	0.8884	0.6763	0.8221	0.8245
**K562**				
ChromNet	**0.9092**	**0.5519**	0.7534	0.7333
ChromNet_onlyCTCF	0.8964	0.5012	0.7332	0.6977
ChromNet_onlyATAC	0.8567	0.4762	**0.7725**	**0.7482**
**GM12878**				
ChromNet	**0.9147**	**0.5698**	**0.8492**	0.7723
ChromNet_onlyCTCF	0.9136	0.5175	0.8465	0.7480
ChromNet_onlyATAC	0.8332	0.4871	0.8324	0.7829
**HCT116**				
ChromNet	**0.8513**	**0.4522**	**0.6477**	**0.6675**
ChromNet_onlyCTCF	0.8480	0.4486	0.6118	0.6420
ChromNet_onlyATAC	0.7818	0.4182	0.6390	0.6582

*Note: Bold values indicate the best performance for each metric.

Table S11. Performance comparison of ChromNet and C.Origami in AML sample predictions. ChromNet was trained with epigenetic features (DNA sequences, CTCF CUT&Tag, and ATAC-seq) from PBMC_103 and AML_270, while the Hi-C contact matrices used for supervision were from PBMC_103 only. Model performance was evaluated on multiple AML patient samples (e.g., AML_018, AML_472, AML_629) and benchmarked against C.Origami, which was trained solely using PBMC_103 epigenetic signals.

Methods	Insul. P.Corr.	Insul. S.Corr.	Dist. P.Corr.	Dist. S.Corr.
		PBMC_103		
C.Origami	0.8505	0.5747	0.6080	0.6454
ChromNet	**0.8651**	**0.6133**	**0.6175**	**0.6516**
		AML_018		
C.Origami	0.2831	0.0910	0.2336	0.1839
ChromNet	**0.7830**	**0.4571**	**0.4571**	**0.5430**
		AML_270		
C.Origami	0.3824	0.2664	0.2882	0.3442
ChromNet	**0.7264**	**0.5380**	**0.4724**	**0.5396**
		AML_472		
C.Origami	0.4096	0.1928	0.3426	0.3693
ChromNet	**0.7879**	**0.5197**	**0.5596**	**0.5929**
		AML_546		
C.Origami	0.7047	0.5219	0.4573	0.5244
ChromNet	**0.7500**	**0.5639**	**0.4651**	**0.5322**
		AML_027		
C.Origami	0.6606	0.3817	0.4199	0.4502
ChromNet	**0.7942**	**0.5196**	**0.5378**	**0.5747**
		AML_168		
C.Origami	0.2832	0.0403	0.1985	0.1761
ChromNet	**0.5722**	**0.3169**	**0.4715**	**0.5122**
		AML_629		
C.Origami	0.6827	0.5136	0.3757	0.3652
ChromNet	**0.7914**	**0.5760**	**0.5508**	**0.6058**

*Note: Bold values indicate the best performance for each metric.

References

[1] The ENCODE Project Consortium, Jill E. Moore, Michael J. Purcaro, Henry E. Pratt, Charles B. Epstein, Noam Shoresh, Jessika Adrian, et al. "Expanded Encyclopaedias of DNA Elements in the Human and Mouse Genomes." Nature. **2020**. 583 (7818): 699–710.
